# Effects of Ring Opening and Chemical Modification on the Properties of Dry and Moist Cellulose—Predictions with Molecular Dynamics Simulations

**DOI:** 10.1021/acs.biomac.4c00735

**Published:** 2024-11-25

**Authors:** Patric Elf, Per A. Larsson, Anette Larsson, Lars Wågberg, Mikael S. Hedenqvist, Fritjof Nilsson

**Affiliations:** † School of Engineering Sciences in Chemistry, Biotechnology and Health, Fibre and Polymer Technology, KTH Royal Institute of Technology, Stockholm SE-100 44, Sweden; ‡ Department of Chemistry and Chemical Engineering, Chalmers University of Technology, Gothenburg SE-412 96, Sweden; § FibRe Centre for Lignocellulose-based Thermoplastics, KTH Royal Institute of Technology, Stockholm SE-100 44, Sweden; ∥ FibRe Centre for Lignocellulose-based Thermoplastics, Chalmers University of Technology, Gothenburg SE-412 96, Sweden; ⊥ FSCN Research Centre, Mid Sweden University, Sundsvall 85170, Sweden

## Abstract

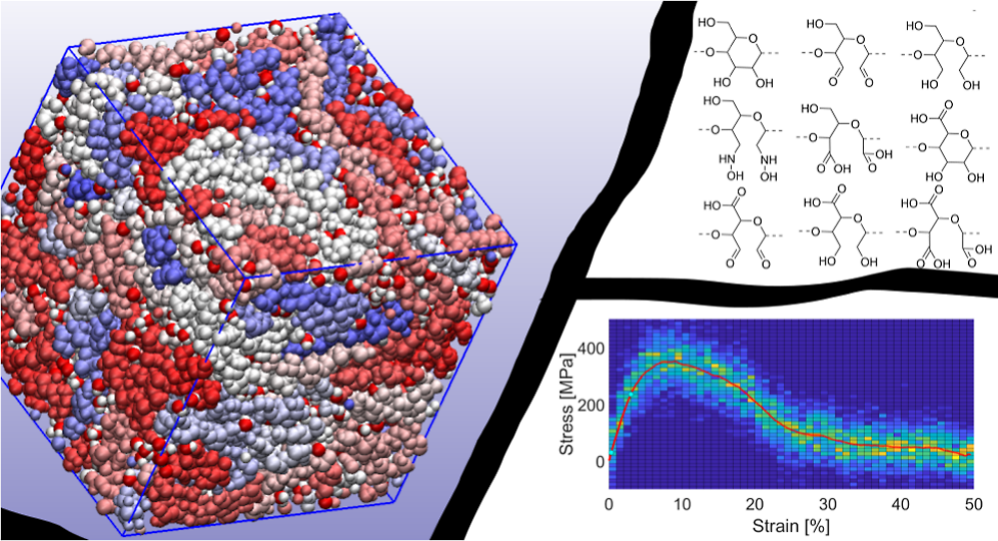

Thermoplastic properties in cellulosic materials can be achieved by opening the glucose rings in cellulose and introducing new functional groups. Using molecular dynamics, we simulated amorphous cellulose and eight modified versions under dry and moist conditions. Modifications included ring openings and functionalization with hydroxy, aldehyde, hydroxylamine, and carboxyl groups. These modifications were analyzed for density, glass transition temperature, thermal expansivity, hydrogen bond features, changes in energy term contributions during deformation, diffusivity, free volume, and tensile properties. All ring-opened systems exhibited higher molecular mobility, which, consequently, improved thermoplasticity (processability) compared to that of the unmodified amorphous cellulose. Dialcohol cellulose and hydroxylamine-functionalized cellulose were identified as particularly interesting due to their combination of high molecular mobility at processing temperatures (425 K) and high stiffness and strength at room temperature (300 K). Water and smaller side groups improved processability, indicating that both steric effects and electrostatics have a key role in determining the processability of polymers.

## 1 Introduction

Modified cellulose materials are promising renewable replacements for fossil-based plastics owing to the natural abundance and availability of cellulose from sources such as trees and other plants.^1−3^ This transition agrees with the United Nations sustainability development goals (SDGs),^1−3^ especially SDG 12, “responsible consumption and production”, assuming that the recycling of the new materials is properly handled.^4,5^ Currently commercially available thermoplastic cellulose derivates include cellulose acetate, cellulose butyrate, ethyl cellulose, and methyl cellulose.^1−3^

For the plastic-producing industries, transitioning to using biobased raw materials poses a significant challenge. For example, if substantial costs in terms of updating production facilities are to be avoided, the new materials should preferably be thermoformable and seamlessly integrate with existing plastic processing techniques. Moreover, the degree of chemical modifications of the native cellulose structure should be minimized.^1−3^ However, despite these boundary conditions, it has become obvious that, for example, EU legislation aims for a transition toward nonfossil-based materials and includes restrictions on single-use plastics such as plastic straws and cutlery. The industry will adapt accordingly with practical solutions and is actively seeking sustainable alternatives to replace these excellent materials with similarly excellent ones that are biobased, renewable, recyclable, and biodegradable. This is a challenging task that also demands the development of new scientific insights into how to tune biobased materials to meet these strenuous demands.

**Figure 1:**
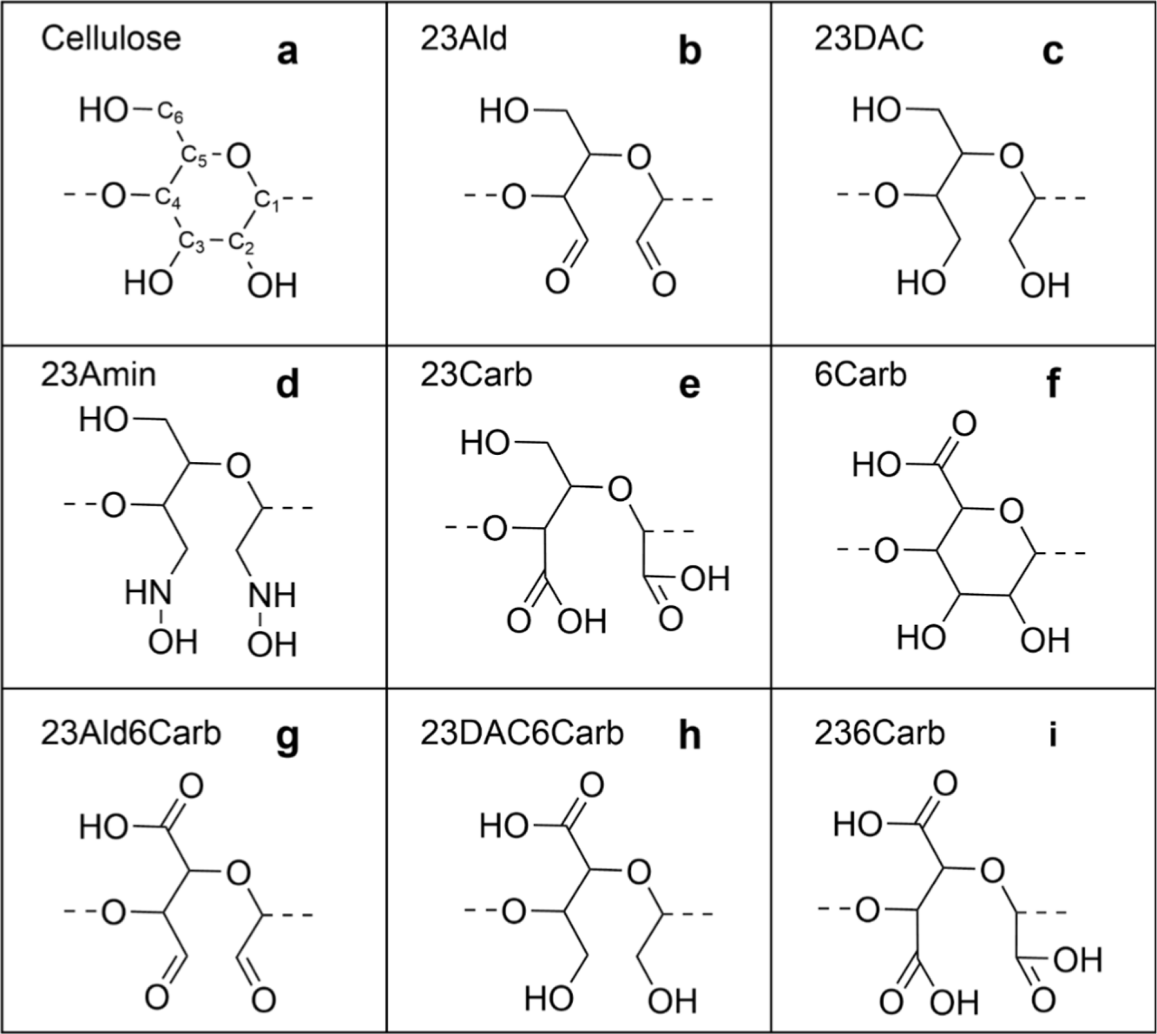
Monomers used with generic names for (a) cellulose, β-1,4 linked glucose, (b) 23Ald, (c) 23DAC, (d) 23Amin, (e) 23Carb, (f) 6Carb, (g) 23Ald6Carb, (h) 23DAC6Carb, and (i) 236Carb.

Recent developments have shown that chemical modification of cellulose can improve its thermoplastic properties, including decreasing viscosity and increasing pliability.^6−8^ The glucose subunits of cellulose have three hydroxy groups that can be substituted and a ring structure that can be opened. Substitution of the hydroxy groups can, for example, yield thermoplastic cellulose esters, whereas opening of the glucose rings can yield dialcohol cellulose.^9^ At high moisture contents, dialcohol cellulose can, for instance, be thermoformed with extrusion, which was also predicted by molecular dynamics (MD) simulations.^10^ As described by López Durán et al., there are several ways to potentially achieve a ring-opened cellulose with the hydroxy groups replaced by other functional groups (Figure 1).^11,12^ Their experimentally demonstrated modifications were used as a basis for this study, in which modifications of fully amorphous systems were used. In their work, they describe the formation of an amorphous shell of modified cellulose surrounding the core of crystalline cellulose fibrils. This suggests that if the degree of modification can be increased or the amorphous layers separated, these materials could offer innovative opportunities for the development of cellulose-based, isotropic thermoplastic, or thermoelastic materials. As these modifications have already been shown to be possible through relatively simple oxidation/reduction steps, they are good candidates for further investigation. By choosing the same modifications as in López Durán et al.,^11,12^ we also got the possibility to verify our predictions with experimental data. In addition to the thermoformable dialcohol cellulose, it is possible that one or several of the other modifications also yield a cellulose material with improved thermoformability as well as improved mechanical properties. To maintain the fiber structure would naturally be valuable since it would circumvent the need for large volumes of solvents in combination with an ease of handling and, possibly, a decreased overall energy demand for the processing. The purpose of this work is to use MD simulations to predict whether any of the suggested routes in Figure 1 have this potential—to improve thermoformability and mechanical properties.

A notable advantage of MD is its ability to assess the influence of factors such as chemical structure without concomitant change in other variables that impact ductility, such as molecular weight, plasticizer and moisture content, temperature, pH, molecular interactions, morphology, and crystallinity.^10,13−18^ Native cellulose-based materials have complex hierarchical structures and typically encompass both crystalline and amorphous (disordered) regions, but purely amorphous cellulose can also be obtained.^19−23^ A pronounced lack of order is typically advantageous from a polymer processing standpoint due to its tendency to provide a softening of the material, facilitate isotropic material properties, and improve the conversion efficiency.^15,24−26^ The last effect is attributed to the comparatively limited accessibility of the crystalline regions for chemical modifications. To highlight the effect of chemical structure rather than morphological features, our MD simulations were thus applied to fully amorphous cellulose-based systems. Isobaric volume–temperature data was produced to assess glass transition temperature, density, and thermal expansivity of cellulose and of ring-opened and substituted systems (Figure 1). Systems equilibrated at 300 and 425 K were also evaluated to assess the mechanical properties, hydrogen bond features, different energy contributions, and free volume. The lower temperature represents room-temperature conditions, and the higher temperature represents a processing temperature used to extrude cellulose derivatives.^27,28^

## 2 Methods

### 2.1 Systems

Nine different cellulose-based MD systems, with water contents ranging from 0 to 20 wt %, were constructed at 300 and 425 K. Monomer repeat units, presented in Table 1 and Figure 1, were prepared using Biovia Materials Studio (2016). The basis for all monomers was d-glucose units with the C_1_ and C_4_ carbons connected via β-1,4 glycosidic bonds. This base monomer, shown in Figure 1a, was then modified to create additional monomers, shown in Figure 1b–i, by altering the hydroxyl groups on C_2_, C_3_, and C_6_, as well as breaking the bond between C_2_ and C_3_. A summary of the modifications to the d-glucose monomer is provided in Table 1.

**Table 1:** Summary of System Ring Openings (ROs) and Modifications

sys no.	name	RO	modifications
a	cellulose	no	none
b	23Ald	yes	aldehyde at C_2_ and C_3_
c	23DAC	yes	hydroxy group at C_2_ and C_3_
d	23Amin	yes	hydroxylamine on C_2_ and C_3_
e	23Carb	yes	carboxylic acid group on C_2_ and C_3_
f	6Carb	no	carboxylic acid group on C_6_
g	23Ald6Carb	yes	aldehyde at C_2_ and C_3_ and carboxylic acid group on C_6_
h	23DAC6Carb	yes	alcohol at C_2_ and C_3_ and carboxylic acid group on C_6_
i	236Carb	yes	carboxylic acid group at C_2_ and C_3_ and on C_6_

Polymers of 36 monomers and three monomer long oligomers were created with each monomer a–i, all in their protonated form. All polymer chains were terminated with hydroxy groups (–OH), either by adding a hydrogen atom to an oxygen terminal or by adding a hydroxyl group to a carbon terminal.

The oligomers with three monomers were uploaded in Charmm-Guís ligand builder to generate Charmm36 force field parameters and partial atomic charges.^29,30^ The parameters and charges for these were then split into three residues: the start, middle, and terminal. They were then compiled, added to a residue topology file, and implemented with a Charmm36 force field.

For each of the nine residue types, molecular systems consisting of 30 polymer chains, each 36 monomers long, with 1 starting, 34 middle, and 1 terminal monomer, were constructed using GROMACS.^31^ These systems were further supplemented with 0, 5, 10, or 20 wt % water molecules. The TIP3P water model, which has been shown to work reasonably well in carbohydrate systems, was used.^32−34^ There are limitations to the TIP3P water model which must be taken into consideration, such as its inability to capture bimodal tetrahedral order distribution with carbohydrates, as well as its tendency to aggregate at low concentrations, which in turn can lead to increased diffusivity of both the water molecules and polymers.^35,36^ However, since TIP3P is computationally very efficient and still gives reasonable results, especially when focusing on comparisons between different systems rather than on absolute values, it was regarded as an appropriate choice of water model for this study. The amorphous systems were equilibrated for a total of 19.25 ns using a modified version of a slow-decompression scheme, which has previously been shown to effectively equilibrate even stiff molecular systems.^17^ The scheme iterates between high and low temperatures with gradually increasing pressure, followed by a slow decompression phase at a lower pressure. The slow-decompression scheme was modified by adding an initial step with low pressure (1 atm) and moderately high temperature (500 K), as shown in Table SI1. This modification enhances the stability of the simulations as the lower starting temperature reduces the initial energies and velocities of the molecules. The initial simulation box is large (20 × 20 × 20 nm^3^) with low density, but during the equilibration process, which cycles through high and low temperatures and pressures, the box gradually shrinks and the density increases. As a result, the water molecules move freely throughout the computational domain during the initial stages of the equilibration but become more confined as the box shrinks and the temperature decreases. This is exemplified in Figures SI1–SI3, which show the movement of a single water molecule throughout the entire 21-step equilibration process, and in Figure SI4, which presents five snapshots from the final 10 ns of equilibration for unmodified cellulose with 5 and 20 wt % water, respectively. Figures SI5 and SI6 show that the total energy and density remain mainly stable during the final 10 ns of the equilibration. These equilibrated systems, containing 30 polymer chains with 36 monomers each, were subsequently used as starting configurations for all production simulations.

### 2.2 Simulation Details

For all the simulations, a Verlet cutoff scheme^37^ was used for the neighbor search, and for the electrostatic interactions, a fourth order particle mesh Ewald summation was used for long-range electrostatics, with a Coulomb cutoff of 1.2 nm. For the van der Waals interactions, similar to the electrostatic interactions, a cutoff distance of 1.2 nm was also used.

#### 2.2.1 Density

Densities and specific volumes of polymer systems at temperatures (*T*) between 150 and 600 K were obtained from isothermal–isobaric (*NPT*) simulations. After the previously mentioned slow-decompression scheme, the equilibrated initial systems were further equilibrated for 30 ns either at 600 K (for the wet systems) or at 800 K (for the completely dry and thus less mobile systems). Thereafter, the systems were slowly cooled in increments of 25 K (each step for 10 ns) down to 150 K. Parrinello–Rahman^38^ pressure coupling with 1 atm of pressure was used, and the density at each temperature was computed as an average over the last 0.5 ns of the simulation. The drift in the system, which was calculated from box fluctuations, was monitored over the entire 10 ns simulation time of each temperature but is only presented for the final 0.5 ns, from which the density calculations were performed, which is long enough for the systems to stabilize, which can be seen exemplified in Figures SI7 and SI8. The density at 300 K was also computed from the last 0.5 ns of the initial *NPT* equilibration simulation.

#### 2.2.2 Glass Transition Temperature

Broken stick regression is a commonly used method for finding *T*_g_, where the specific volume of a material is plotted as a function of temperature, and the data is fitted with two lines using data points clearly above and below *T*_g_, respectively. The intersection of the lines is an estimate of *T*_g_.^10,18^ In this study, the six low (150–275 K) and six high temperatures (475–600 K) from the cooling curves described in Section 2.2.1 were used for the fitting. The reason for including also very high temperatures is that the linear regression requires enough data points above *T*_g_ for all materials and moisture contents. Even though the highest temperatures are above the boiling point of water as well as above the degradation temperature of cellulose, the simulations are still viable. The reasons are that since the force field of the simulation does not allow bond breaking, there will be no degradation, and since the simulated bulk materials are not in contact with, e.g., air, no evaporation of moisture will occur.^39,40^

#### 2.2.3 Thermal Expansion Coefficient

The volumetric thermal expansion coefficient α_V_(*T*) was calculated by using eq 1, with two densities ρ(*T*) inserted from the cooling curves of Section 2.2.1. The specific volume is *V*(*T*) = 1/ρ(*T*), and the example in eq 1 is for the temperature range 300–325 K. Due to the strongly nonlinear character of α_V_(*T*), a relatively narrow temperature interval is recommended. Consequently, a temperature interval of 25 K was used.
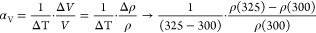
1

#### 2.2.4 Hydrogen Bonds

Hydrogen bonds were defined as when the distance *r*_HB_ between acceptor oxygen and donor oxygen was less than 0.35 nm, and the angle θ_ΗΒ_ (between hydrogen–donor oxygen–acceptor oxygen) was less than 30°. *NVT* simulations (10 ns long) were performed for all nine systems at 300 and 425 K. The hydrogen bond lifetime and half-life were evaluated using the existence function *C*_HB_ (eq 2), available as a built-in function in GROMACS. Since the hydrogen bond lifetime tended to be much longer than 10 ns, the existence function data was fitted to a weighted decay function with two terms (eq 3; *K*_1_, *K*_2_, τ_1_, and τ_2_ are fitting parameters), which enabled an extrapolation and integration of the existence function (eq 4) until it reached a value of 0 and 0.5 for the hydrogen bond lifetime and hydrogen bond half-life, respectively. This was done as these are quite rigid systems, and many bonds never break, which means that the hydrogen bond lifetime, in essence, becomes infinite, not giving much to interpret. Hence, the half-life is more meaningful.
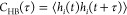
2
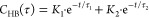
3
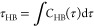
4

#### 2.2.5 Deformation Properties

Semi-isotropic deformation simulations were performed at both 300 and 425 K. The deformations in the two directions perpendicular to the main deformation direction were coupled so that they were forced to be equal. Parrinello–Rahman pressure coupling at 1 bar and velocity-rescale temperature coupling were used in the deformation simulations. The stress σ(ε) was determined as σ(ε) = −*P*_*z*_(ε), where *P*_*z*_ represents the smoothed pressure tensor in the *z*-direction. The strain ε was defined as ε = (*L*_t_ – *L*_0_)/*L*_0_, where *L*_t_ denotes the extended box length in the *z*-direction and *L*_0_ represents the initial box length. Total strain interval ranged from 0% to 100%, and the deformation rate was set to 0.001 μm/ns. The stress–strain curve was smoothed using a strain range ±2.5% around each strain data point. To obtain a smooth curve with zero stress at zero strain, the stress–strain data was reflected, with a negative sign, around ε = 0.

Young’s modulus *E* was calculated from Hooke’s law (*E* = σ/ε) in the strain interval 0.3% to 3%, and maximum tensile strength was calculated as the largest smoothed stress between 3% and 97% strain. The deformation simulations were performed in all three orthogonal directions (*X*, *Y*, and *Z*) using the same initial configurations. This standard procedure enabled an error estimate of the mechanical properties.

Free volume was calculated as a function of the deformation of the system using three different spherical probe sizes with a radius of 0.1, 0.05, and 0.01 nm. In parallel, the number of hydrogen bonds was also determined by using GROMACS built-in functions.

Poisson’s ratio (ν) for an applied strain in the *z*-direction was calculated from eq 5 using a strain interval of 1 to 2% strain.
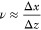
5

As the simulated systems were isotropic, bulk modulus (*K*) was calculated with *E* and ν using Lame’s relation (eq 6).^39^
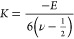
6

#### 2.2.6 Diffusion

Mean square displacement (MSD) was used to determine the diffusivity and mobility of both the water molecules and the polymer chains in 10 ns *NVT* simulations. Diffusivity was calculated for the center of mass of each individual polymer chain using the GROMACS built-in function and then averaged, yielding the standard deviation (STD) as an error estimate. Mean square displacement was also calculated for all atoms in the polymer chain, from which a least-square fit was made for the linear part of the mean square displacement curve, between the 1 and 9 ns part of the 10 ns simulation, as seen in Figure SI9. Diffusivity for the water molecules was calculated similarly using the center of mass and a least-squares fit.

## 3 Results and Discussion

The simulation results are structured into three sections: (1) *NPT* properties, including density, specific volume, and thermal expansion coefficient as a function of temperature and *T*_g_ determined with broken stick regression; (2) *NVT* properties, including diffusion and hydrogen bond analysis; and (3) deformation properties, including stress–strain relationships, elastic modulus, maximum tensile strength, bulk modulus, energy contributions within the system, and free volume as a function of strain. The bulk modulus and thermal expansion properties are particularly important for materials being hot-pressed and during thermoforming if the mold does not allow the material to flow out. The thermal expansion coefficient is also an important factor for laminates and composite materials as they will need to deform uniformly with the materials they are used with.

### 3.1 *NPT* Simulation Properties

**Figure 2:**
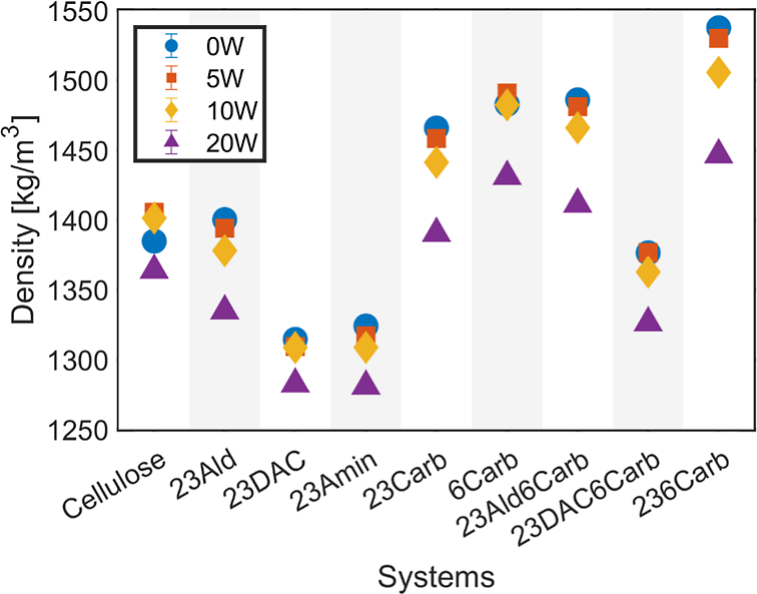
Density of equilibrated systems at 300 K with relative STD < 1%.

The densities at room temperature (300 K) for all nine polymer systems with 0–20 wt % moisture contents are presented in Figure 2. This data is for systems initially equilibrated directly at 300 K. Note that the standard deviation (STD) of the density, due to fluctuations within the simulations over the sampled time, was less than 1%. The dialcohol cellulose and the aminated cellulose system, both ring-opened, showed a density significantly lower than that of native cellulose. The decrease was similar to that for cellulose acetate but smaller than that for ethyl cellulose.^24,40^ In the two latter systems, the decrease was due to steric effects from the bulky side groups.

For the simulated ring-opened systems here, a decrease in density was observed when introducing water, with a significant drop at the 20 wt % water content (Figure 2 and Table SI2). However, this was not the case for the nonring-opened systems (cellulose and 6Carb). In these two systems, the addition of 5 and 10 wt % of water filled up the free volume between the rigid polymer chains, leading to a minor increase in density in parallel with an increase in volume.

The ring-opened aldehyde (23Ald) and the dialcohol carboxylated system (23DAC6Carb) also showed lower densities than the cellulose system at similar water contents, both under dry and moist conditions. Due to the larger and denser groups, the carboxylated systems had a higher density, with the one with three carboxyl groups (236Carb) having the highest density. Among the carboxyl-containing systems, the ring-opening decreased density, especially noticeably for the dialcohol system (23DAC6Carb). It was seen that the cellulose system and the dialcohol cellulose system showed similar density as had previously been reported for simulations, at around 1.4 and 1.3 g/cm^3^, respectively.^10,26^ These simulation values are slightly lower than corresponding experimental values (1.48–1.50 g/cm^3^),^19,41^ which is likely due to the idealized nature of the simulations compared to experiments, as well as due to the fact that the polymer chains were only 36 monomers long, which is shorter than typical cellulose molecules in nature.

When the simulated densities are compared with corresponding experimental data from Lopez Duran et al.,^12^ the densities of most polymers fall within approximately the same range (ca. 1.4–1.5 g/cm^3^). Furthermore, the 23Amin system exhibits the lowest density in both the simulated and the experimental systems. However, the differences between the simulated materials are larger than those between the experimental materials. This discrepancy is likely due to the fact that the simulated materials are fully modified and fully amorphous, whereas the experimental materials are only surface-modified, leading to smaller differences in density compared with the unmodified cellulose reference.

In Table SI2, it is shown that the approximate increase in volume of the systems with increasing water content tends to be in the same range for all systems, further cementing the idea that density differences among the systems are due to the introduction of additional atoms/groups that increase mass and are not a result of electrostatics or of the introduction of more potential hydrogen bonds which could form.

As for the completely dry materials, there was a deviation in density between systems initially equilibrated directly at 300 K and systems equilibrated at 800 K and cooled to 300 K, as seen in Figure SI10a. This is because the systems and polymer chains entered a liquid-like, viscous state at 800 K and thus, with the extra mobility offered, were free to move and expand to some extent. Comparatively, from the equilibration process, when the system is iterated through temperature differences and high pressures to conform to a densely equilibrated state, it becomes locked in a more rigid structure with more free volume after slow cooling. These locked-in lower-density states could possibly form in vacuum, but in a real scenario, due to osmotic pressure, the water in the air (humidity) would likely enter such voids. This is especially the case as water, at least the first percent of it, tends to bind strongly to cellulose and cellulose derivatives, being also difficult to remove completely due to the hydrophilic nature of the hydroxy groups.^42−44^ For the more mobile materials with 5–20 wt % moisture, the densities obtained with the two different techniques coincided (Figure SI10a–d).

**Figure 3:**
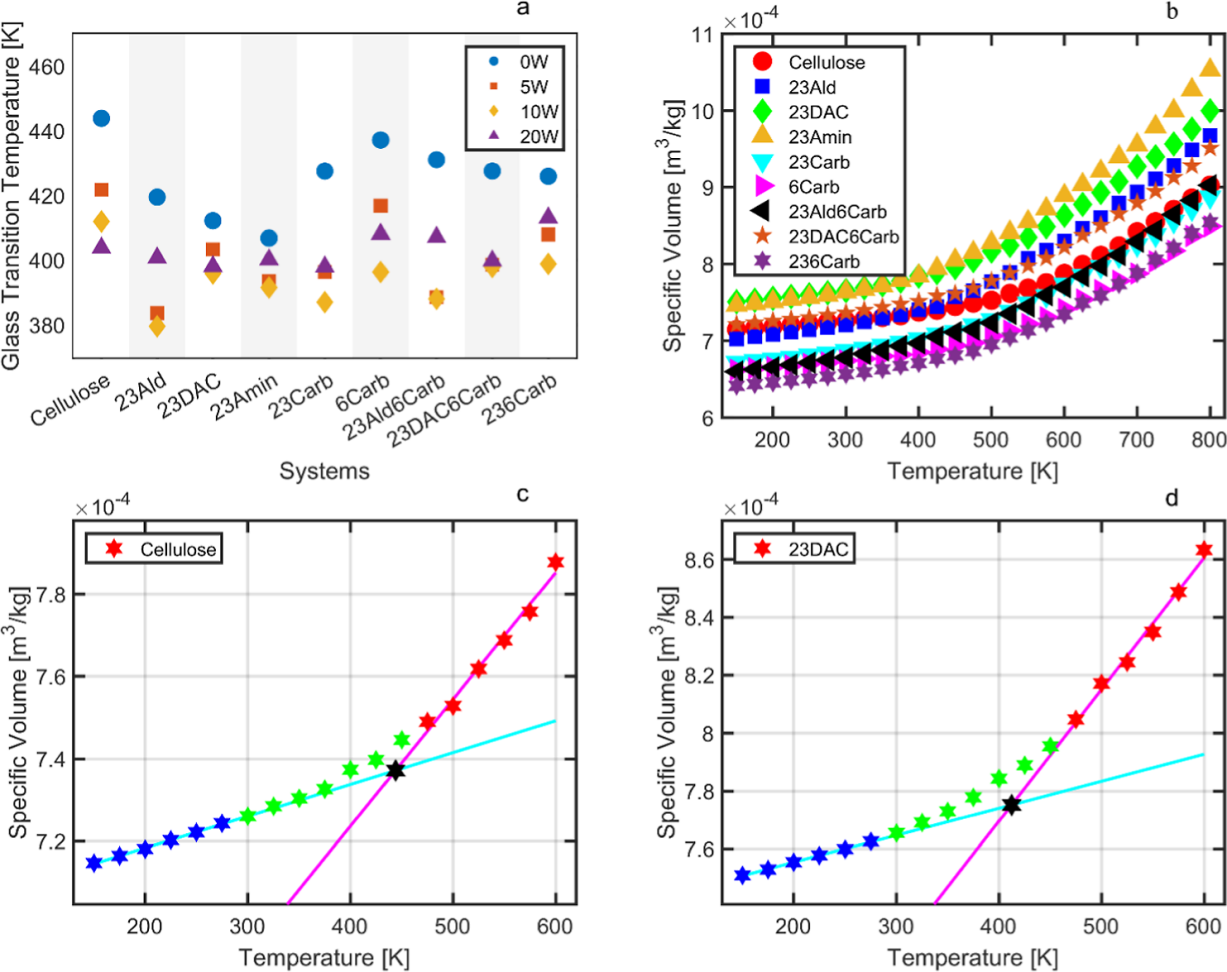
(a) *T*_g_ of the systems; (b) *PVT* curves for all dry systems; (c) *PVT* curve for the dry Cellulose system, including linear fits and intersection marked; and (d) *PVT* curve for the dry 23DAC system, including linear fits and intersection marked with a black star, commonly used to estimate *T*_g_. In (c,d), the colors represent different intervals, where blue is the data points used for the lower temperature regression, green is the points unused, and red is the points used for the high temperature regression.

The *T*_g_s of the different structures, determined with broken stick regression, are summarized in Figure 3a, as well as summarized in Table SI3, as well as the drift in %/ns during the sampling interval in Table SI4 for the systems. All *T*_g_ values were derived from curves with a specific volume as a function of temperature, as exemplified in Figure 3b. Two linear regressions for the dry cellulose and 23DAC systems are presented in Figure 3c,d. The impact of the low molecular weight and degree of polymerization in the simulations, however, raises questions as to how this affects the resulting *T*_g_ as it is well-known that *T*_g_ is dependent on the molecular weight. With a molecular weight of roughly 6000 u for the polymer chains, we can, from comparing with literature data, determine that this would only underestimate the *T*_g_ by a few degrees, roughly 10–20 °C, as with similar amorphous polymers such as polystyrene.^45−47^ Some of the dry systems had *T*_g_ values above the deformation simulation temperature (425 K), but since the transition is gradual, the chosen temperature should still be acceptable.

An observed trend was that ring openings overall tended to decrease *T*_g_, which may be attributed to the increased mobility of the polymer systems.^48^ We see in Figure 3a that the cellulose and 6Carb systems, the systems that still have a ring structure, had high estimated glass transition temperatures for all water contents, showing the importance of ring openings for decreasing *T*_g_. Apart from making the polymers more mobile, which, of course, is also related to the softening and melt temperatures of the polymers, it also increases the mobility of the functional groups, allowing them to interact more freely with other polymer chains, solvents, or molecules such as plasticizers. Amination of the ring-opened systems, as exemplified in system 23Amin, resulted in a further decreased *T*_g_ compared with dialcohol cellulose ring openings (23DAC). Systems containing hydroxy groups exhibited slightly lower *T*_g_ values compared to those with carboxyl groups, which, in turn, demonstrated lower values than systems with aldehydes, thus establishing the order of hydroxy < carboxyl < aldehyde in terms of *T*_g_. The inclusion of 5–10 wt % water resulted in a decrease in *T*_g_ for all materials. However, at even higher moisture contents (20 wt %), all materials, except pure cellulose, exhibited a subsequent increase, consistent with findings in literature.^44^ This increase is probably an artifact resulting from the saturation of water in the systems, given that 20% water represents quite a high amount, roughly 2–3 water molecules per repeating unit of the polymer (see Table SI5). At sufficiently high water contents, the simulated glass transition temperatures of all polymers will gradually converge to a common value, determined by the specific volume of pure water.^44^ A large number of water molecules per repeat unit was expected to correlate positively with a low *T*_g_, but only a weak tendency was observed (Table SI5 and Figure 3a).

**Figure 4:**
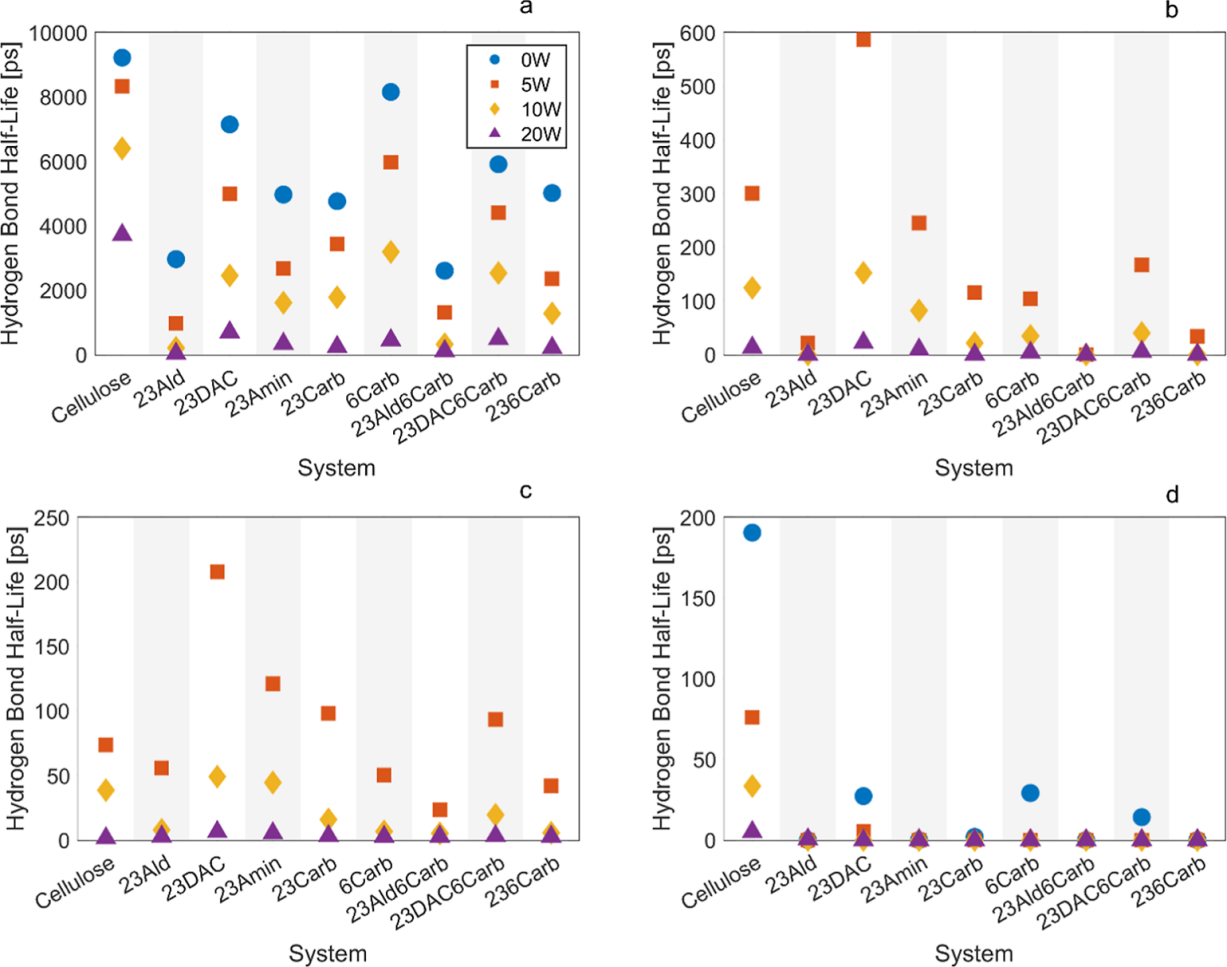
Hydrogen bond half-lives between (a) polymer–polymer at 300 K, (b) polymer–water at 300 K, (c) water–water at 300 K, and (d) polymer–polymer at 425 K.

**Figure 5:**
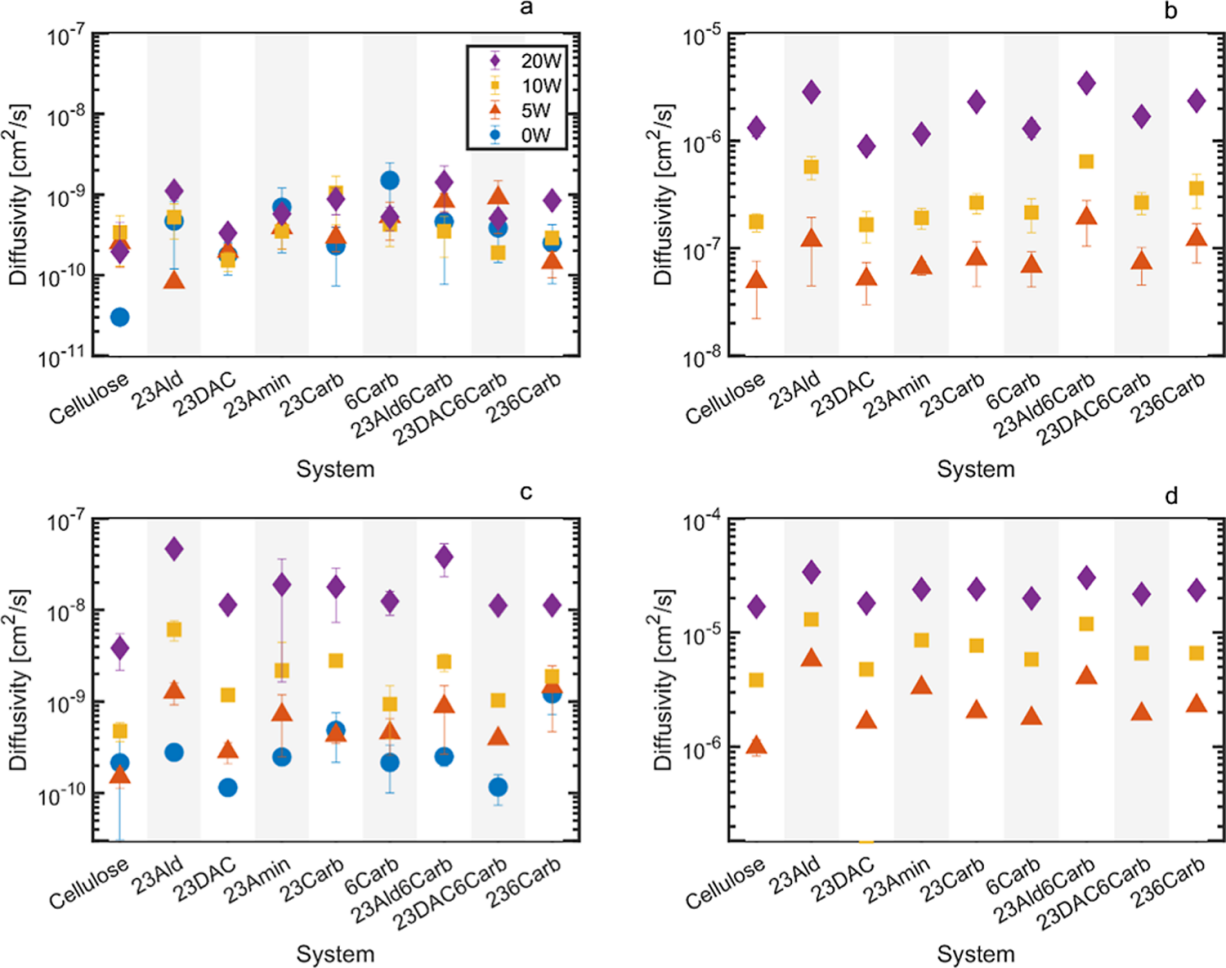
Diffusivity calculated using mean square displacement of all atoms for (a) polymer at 300 K, (b) water at 300 K, (c) polymer at 425 K, and (d) water at 425 K.

The thermal expansion coefficient α_V_ displayed significant variation among the systems, particularly at low moisture contents (see Table 2). Cellulose exhibited the lowest α_V_ values. Generally, α_V_ increased with the moisture content, although the trend was not consistently linear. In several systems, the rise in α_V_ became more pronounced beyond 5–10 wt % moisture, likely attributed to saturation effects. At 20 wt % water, the systems with aldehyde modifications, 23Ald and 23Ald6Carb, exhibited the highest α_V_ values. This suggests that the nonlinearity may be attributed to the saturation of hydrogen bond acceptors in the polymer chains, thereby magnifying the impact of adding additional water molecules. This hypothesis is further supported by the results for water–polymer hydrogen bond half-lives (Figure 4) and diffusivities (Figure 5), where we see that hydrogen bond half-life decreases substantially for all systems and approaches zero for the 20 wt % water systems, while for 23Ald and 23Ald6Carb, it is already close to nil at 5 wt % water, while the diffusivity for water remains the highest for these systems. It is important to note that water diffusivity is still 2 orders of magnitude lower than the self-diffusivity of the TIP3P water model at 300 K, even for the systems with the most mobile water, meaning that the water is not unobstructed and is significantly clustered.^34,49^

**Table 2:** Thermal Expansion Coefficients (×10^–4^, 1/K)

systems	0 W	5 W	10 W	20 W
cellulose	1.34	1.36	1.57	3.55
23Ald	2.55	2.13	5.12	6.23
23DAC	1.91	1.83	2.78	5.25
23Amin	1.80	2.77	3.56	5.35
23Carb	1.82	3.00	3.01	5.20
6Carb	1.41	2.07	2.40	5.55
23Ald6Carb	2.90	2.91	4.51	6.69
23DAC6Carb	1.90	3.12	2.26	4.82
236Carb	1.67	2.80	3.26	5.71

### 3.2 *NVT* Simulation Properties

Hydrogen bond half-lives and lifetimes for all systems are shown in Figures 4 and SI11, respectively. Generally, hydrogen bond half-life decreased with increasing water content in all interactions: polymer–polymer, polymer–water, and water–water. This can be attributed to water acting as a plasticizer, enhancing system mobility and increasing the likelihood of hydrogen bond breakage. Additionally, hydrogen bond half-life decreased significantly with increasing temperature as higher temperatures lead to greater atomic energy. This increased energy is expressed through enhanced motions within the polymer system, such as vibrations, torsions, and increased molecular velocity, which results in higher molecular mobility. Polymer–polymer hydrogen bond half-lives dropped approximately two magnitudes from 300 (Figure 4a) to 425 K (Figure 4d), resulting in some values close to zero at 425 K. Similar trends were observed for water–water interactions (Figure SI11).

Hydrogen bond half-lives and lifetimes consistently decrease with increasing polymer mobility owing to heightened molecular motion and reduced molecular cohesion. Therefore, they serve as useful indicators when examining polymer processability of amorphous cellulosic systems using MD, particularly since they are less subjective than glass transition temperatures calculated using the broken stick regression method. Hydrogen bonds are relatively weak interactions compared to covalent bonds. The dissociation energy is around 300–400 kJ/mol for covalent C–C bonds and around 17–30 kJ/mol for a hydrogen bond in cellulose.^50−52^ However, due to the sheer number of hydrogen bonds, they have a non-negligible impact on the systems. Adding more water molecules will cause more hydrogen bonds to form among the water molecules themselves as well as between the water and the polymer chains. This reduces the number of bonds between the polymer chains, and since the system becomes more mobile, the overall lifetime of the bonds will decrease. For all systems, the number of hydrogen bonds per monomer at 300 and 425 K was quantified, as shown in Tables SI6 and SI7, respectively.

Aldehyde systems (23Ald and 23Ald6Carb) typically exhibited the shortest hydrogen bond half-lives for all interactions due to the nature of aldehyde groups, which are only able to act as acceptors. Combined with their high diffusivity and low *T*_g_, these materials are promising from a processing perspective, although consideration needs to be taken regarding the fact that the aldehyde groups are likely to cross-link in an actual system. This is due to the reactive nature of the aldehydes forming hemiacetals between the unmodified hydroxy group at the C_6_ carbon and the aldehydes at the C_2_ and C_3_ carbons. These cross-linkages tend to increase the strength of the material, which, in turn, hampers processability as the aldehyde content in a 23Ald system can decrease by over 30% within 2 weeks, presumably through hemiacetal formation.^11,12,53−56^ The systems with intact glucose rings (cellulose and 6Carb) had the longest half-lives, followed by dialcohol celluloses (23DAC and 23DAC6Carb).

Polymer diffusivity increased with increasing temperature and water content, as seen in Figure 5a,c, with a few exceptions for the least mobile systems.^57^ In general, the reliability of diffusion data is higher for the more mobile systems, and since the polymer systems at 300 K were nearly immobile even with added moisture, the self-diffusivities at 425 K are more trustworthy.^58,59^ Note that at 300 K, and to some extent at 425 K, the polymer systems are in a solid state with polymer diffusivity close to zero. This is evident from the mean square displacement (MSD) plots in Figure SI9, where the polymers sometimes fail to establish a true linear regime. The error estimates in Figure 5 are based on the difference between the first and second halves of the MSD curve sampling interval, which is used to calculate the diffusivity. The large differences in error in both Figure 5a,c can be attributed to small random deviations in mobility between the different chains in the nearly immobile systems. In Figure 5b, the larger errors at 5 wt % water compared to higher moisture contents are due to its lower water mobility. The relative errors are larger at this moisture content, but this does not necessarily imply a larger absolute error. In any case, at most temperatures and moisture contents, cellulose exhibited the lowest diffusivity of all of the systems. This was also anticipated since the intact glucose rings in cellulose are bulky and rigid, providing a lower diffusivity compared to ring-opened cellulose derivatives. The other system with intact glucose rings, 6Carb, had a surprisingly high polymer diffusivity at 300 K and 0 wt % water, but this may be due to the bulky nature of the carboxyl group causing higher mobility within the system. But due to the generally low diffusivity of all the polymers at 300 K, it may just be an artifact of the low mobility. The aldehyde systems, 23Ald and 23Ald6Carb, typically exhibited the highest diffusivities among all of the systems, indicating a potentially good processability of these materials. The aldehyde groups would, however, be likely to form cross-linkages under real conditions, hampering processability, which, of course, would need to be taken into consideration and mitigated. In contrast, the dialcohol-based systems (23DAC and 23DAC6Carb) had the lowest diffusivities among all the ring-opened systems. This is explained by the ability of the small groups to rotate and to allow the hydroxy groups to orient themselves in order to retain their electrostatic/hydrogen bond interactions rather than having the entire active group moving. This ensures the preservation of intermolecular interactions while facilitating molecular mobility within the system.

Water diffusion in the cellulosic materials was significantly impeded, being several orders of magnitude lower than the corresponding diffusivity of pure water, which is 2.3–2.6 × 10^–5^ cm^2^/s between 25 and 30 °C,^59^ as seen in Figure 5b,d. This was expected because it is challenging for liquids to diffuse through tightly packed systems due to electrostatic forces, hydrogen bonding, and steric hindrance. Water diffusivity increased with increasing temperature, and the moisture content was typically lowest for cellulose and highest for the aldehydes, in agreement with the polymer diffusivity results. Note that the used water model (TIP3P) is known to systematically overestimate water diffusion coefficients, so the trends are more reliable than absolute diffusiveness.^34,58^ The diffusion and hydrogen bond calculations were performed in the *NVT* ensemble. In the stiff polymer systems, the *NVT* ensemble enabled a higher computational efficiency as compared to the *PVT* ensemble while still providing sufficient accuracy for the diffusion and hydrogen bonding calculations. Given the stiffness of the systems, the choice of the *NVT* or *NPT* ensemble is expected to have only a minimal effect on the diffusion coefficients and the hydrogen bond half-lives.

### 3.3 Mechanical Properties

**Figure 6:**
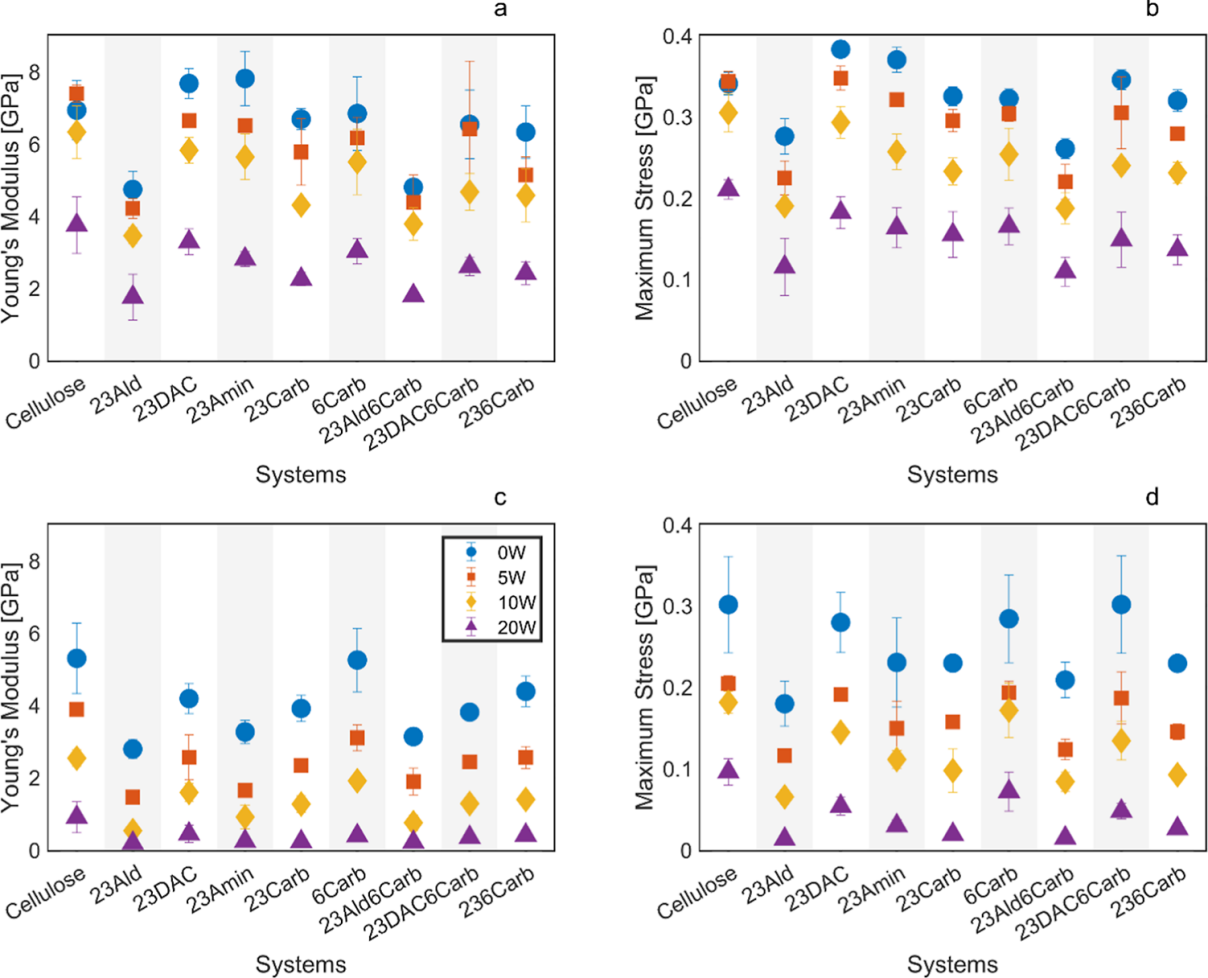
Tensile properties of all systems. (a) Young’s modulus at 300 K, (b) maximum stress at 300 K, (c) Young’s modulus at 425 K, and (d) maximum stress at 425 K.

Young’s modulus and the maximum stress for all systems at 300 and 425 K are plotted in Figure 6. At 425 K, a tendency for both properties to decrease was observed for all ring-opened systems compared to the two materials with intact glucose rings (cellulose and 6Carb). Among the four cellulose-based ring-opened systems (23Ald, 23DAC, 23Amin, and 23Carb), the system with aldehyde groups (23Ald) consistently yielded the lowest values independent of temperature and water content, whereas dialcohol (23DAC) generally exhibited the highest values, often followed by the aminated structure (23Amin), indicating more rigid systems. A high Young’s modulus combined with a low Poisson’s ratio indicates a more rigid and possibly a more brittle material, so analyzing Poisson’s ratio was particularly important for the materials with the highest stiffness, such as 23DAC.^60^ It is important to note that due to the high deformation rates, the values for Young’s modulus and maximum stress are likely to be exaggerated compared to what would be seen experimentally as the rate of deformation for all systems was set at 0.001 μm/ns. Compared to surface-modified fibers, however, Young’s modulus is in a similar range, but it is important to keep in mind that an amorphous structure is likely to behave quite differently from the surface-modified fibrous one.^11,12^ When the modulus data in Figure 6a are compared with experimental results for materials with corresponding modifications,^12^ it is observed that the modulus values are of the same order of magnitude in both cases (2–8 GPa in the simulations versus 2–14 GPa in the experiments). For both simulated and experimental materials, the modulus decreases by 50–75% when 10–20 wt % moisture is added to the initially dry systems. However, in the simulated systems, the comparison is made using a fixed moisture fraction, whereas in the experimental systems, the moisture content is time-dependent and influenced by the water solubility and diffusivity of the individual materials. As a result, the ranking of the modulus values differs between the two cases. For instance, 23Ald shows a comparatively low modulus in the simulations but a high modulus in the experiments as it absorbs only 2.5 wt % moisture, while, for example, 236Carb absorbs 21 wt %. An important conclusion is that the actual moisture content must be carefully controlled when comparing different materials experimentally as water solubility and diffusivity have a significant indirect impact on the mechanical properties of the materials.

Poisson’s ratio for 23DAC was on the same level as that for amorphous cellulose, as seen in Table 3,^61^ indicating that it is not too brittle, which is consistent with experimental findings.^62^ Generally, Poisson’s ratio increased with increasing temperature and water content due to increased molecular mobility. At 300 K and with 0–5 wt % water, all systems exhibited a Poisson’s ratio around 0.3, similar to cellulose *I*_β_ [2 0 0]/[0 0 4],^63^ but at higher temperatures and water content, the differences between the materials became more pronounced. For example, Poisson’s ratios for cellulose and 6Carb at 425 K and with 20% water were 0.40 and 0.49, respectively, which is a large difference and shows the strong increase in molecular mobility when replacing the hydroxy group at C_6_ with a carboxyl group in a water-rich environment. However, it is important to note that the hydroxy group at the C_6_ carbon is not as reactive to modification as the one at the C_2_ carbon, though it may have a higher impact on molecular mobility.^64^ More investigation is required in this regard.

**Table 3:** Poisson’s Constant

system, 300 K	0 W	5 W	10 W	20 W
cellulose	0.30 ± 0.01	0.30 ± 0.02	0.31 ± 0.01	0.34 ± 0.01
23Ald	0.32 ± 0.02	0.34 ± 0.02	0.37 ± 0.05	0.43 ± 0.03
23DAC	0.32 ± 0.01	0.31 ± 0.01	0.35 ± 0.01	0.41 ± 0.01
23Amin	0.29 ± 0.01	0.31 ± 0.00	0.30 ± 0.02	0.39 ± 0.01
23Carb	0.29 ± 0.01	0.30 ± 0.01	0.34 ± 0.01	0.39 ± 0.02
6Carb	0.28 ± 0.01	0.32 ± 0.02	0.32 ± 0.02	0.37 ± 0.01
23Ald6Carb	0.30 ± 0.02	0.32 ± 0.00	0.34 ± 0.03	0.42 ± 0.03
23DAC6Carb	0.29 ± 0.01	0.31 ± 0.02	0.35 ± 0.02	0.41 ± 0.01
236Carb	0.28 ± 0.01	0.32 ± 0.00	0.34 ± 0.02	0.39 ± 0.02

system, 425 K	0 W	5 W	10 W	20 W
cellulose	0.33 ± 0.02	0.35 ± 0.02	0.4 ± 0.00	0.40 ± 0.04
23Ald	0.33 ± 0.03	0.42 ± 0.05	0.45 ± 0.01	0.47 ± 0.02
23DAC	0.33 ± 0.02	0.43 ± 0.03	0.40 ± 0.02	0.48 ± 0.01
23Amin	0.36 ± 0.01	0.38 ± 0.04	0.41 ± 0.04	0.47 ± 0.05
23Carb	0.32 ± 0.01	0.38 ± 0.03	0.42 ± 0.02	0.47 ± 0.06
6Carb	0.32 ± 0.02	0.35 ± 0.03	0.40 ± 0.01	0.49 ± 0.01
23Ald6Carb	0.35 ± 0.04	0.35 ± 0.05	0.41 ± 0.03	0.44 ± 0.04
23DAC6Carb	0.35 ± 0.02	0.37 ± 0.05	0.44 ± 0.00	0.46 ± 0.02
236Carb	0.32 ± 0.02	0.34 ± 0.04	0.40 ± 0.02	0.46 ± 0.01

**Figure 7:**
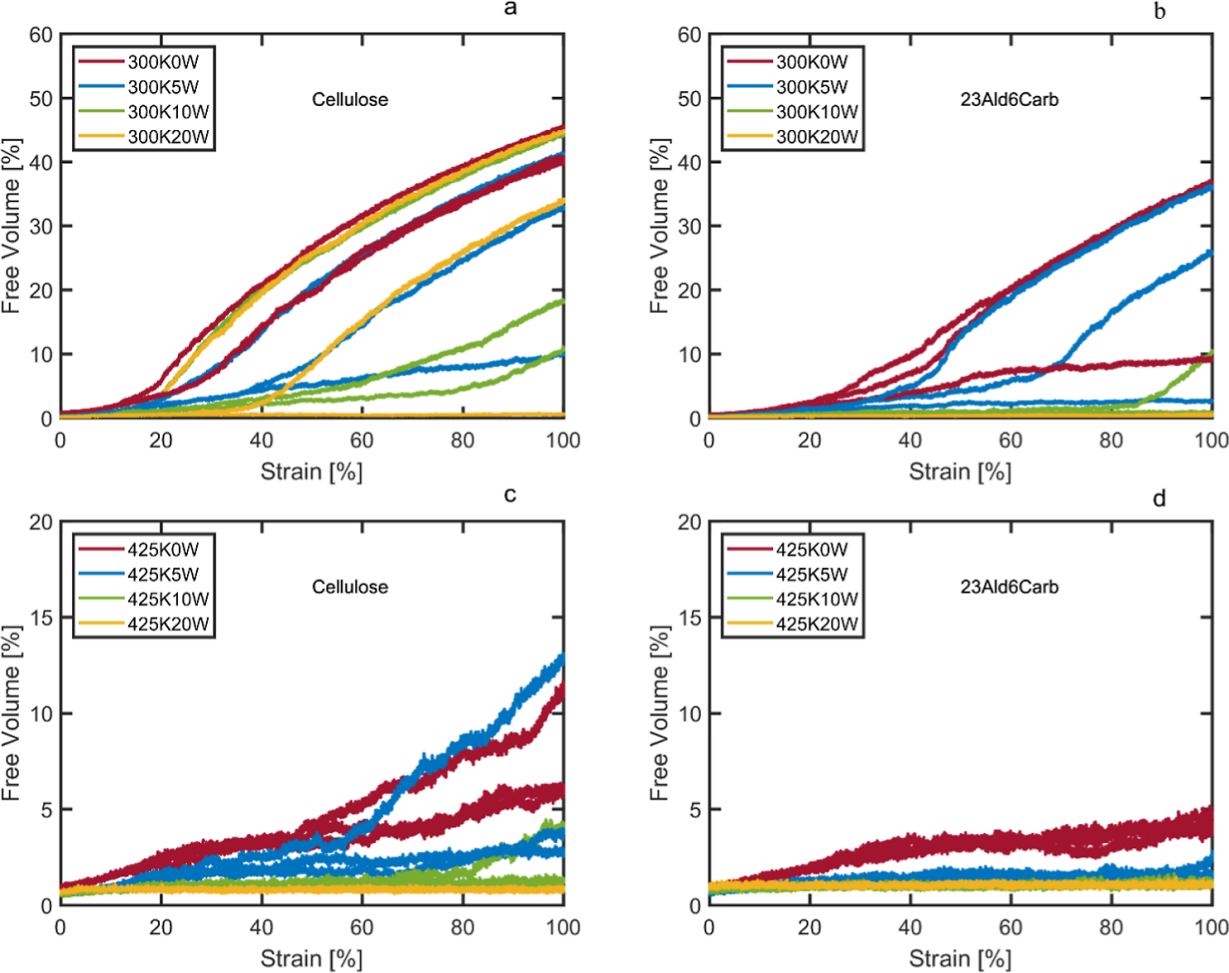
Free volume as a function of strain for (a) cellulose at 300 K, (b) 23Ald6Carb at 300 K, (c) cellulose at 425 K, and (d) 23Ald6Carb at 425 K. A probe size of 0.1 nm was used, and simulations were performed in 3 directions, *X*, *Y*, and *Z*, at each temperature and water content.

For all the systems, especially the dry ones, total hydrogen bond density decreased slightly with increasing strain (0–100%) (Figure SI12). The total number of hydrogen bonds between polymer chains, however, decreased by only a fraction, and the number of bonds between polymers and water decreased even less. The bonds between water molecules were essentially unaffected by the magnitude of the strain, as is also seen in similar works.^65^ The reduced overall hydrogen bond density with strain was, as expected, due to the noncovalently bonded atoms being forced apart as the strain increased. Consequently, the free volume of the system increases as voids form, especially for the more rigid systems, i.e., at lower water content and temperature (Figures 7 and SI13). With increasing moisture content, the fraction of water–water and water–polymer interactions gradually increased compared to polymer–polymer interactions. A significantly reduced total number of hydrogen bonds was observed at 425 K compared to 300 K, indicating that the increased internal energy, molecular mobility, and free volume at elevated temperature decrease the number of potential interactions and the retention rate for hydrogen bonds. We also see a significant difference in the free volume between the differently functionalized/ring-opened systems. This is exemplified by the more rigid cellulose (Figure 7a) and the ring-opened aldehyde and carboxyl functionalized material (Figure 7b). Similar trends were seen for the other ring-opened systems, with the trend being that high temperatures increased the mobility of the polymer chains and decreased the propensity for void formations, the same being the case with increased water content. The 23Ald and the 23Ald6Carb systems were the least likely to form voids, followed by the 23DAC system and the 23Amin system, showing that smaller groups made the systems more flexible and more resistant to tearing, while for 6Carb, the large carboxyl groups, having a ring structure, did the opposite.

**Figure 8:**
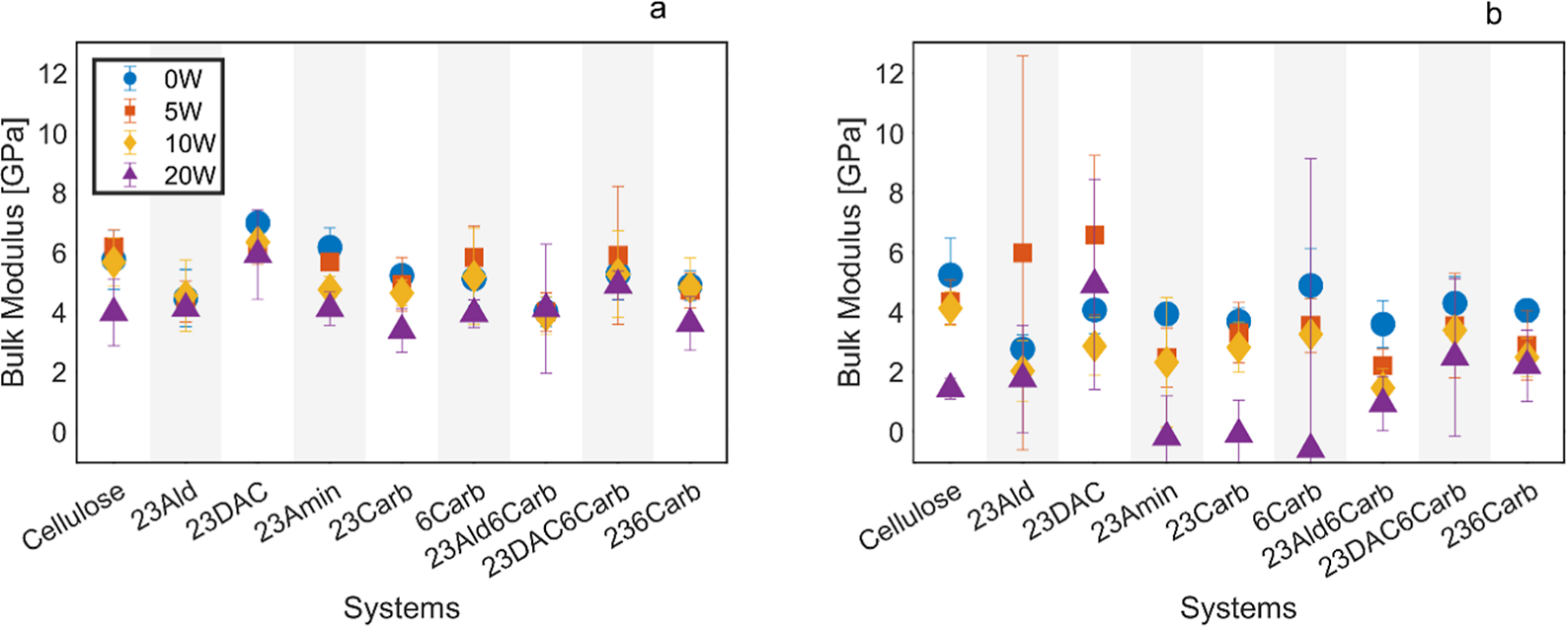
Bulk modulus at (a) 300 and (b) 425 K.

The bulk modulus for all samples was calculated using eq 6, and the results at 300 and 425 K are presented in Figure 8a,b, respectively. At room temperature with 0–10 wt % water, the bulk modulus was highest for dialcohol cellulose (23DAC), closely followed by cellulose and the aminated cellulose derivative (23Amin), and lowest for the aldehyde-terminated derivative (23Ald). Water consistently decreased the bulk modulus with increasing water content by acting as plasticizers, as well as with the lower compressibility of the actual water being roughly 2 GPa at room temperature.^66^ Similar trends were observed at 425 K. However, some systems with high temperatures and water contents exhibited very large STDs. This was attributed to their Poisson’s ratios being close to 0.5 (see Table 3), making the bulk modulus highly sensitive to small natural fluctuations in simulation box size as the denominator in eq 6 approaches zero.

**Figure 9:**
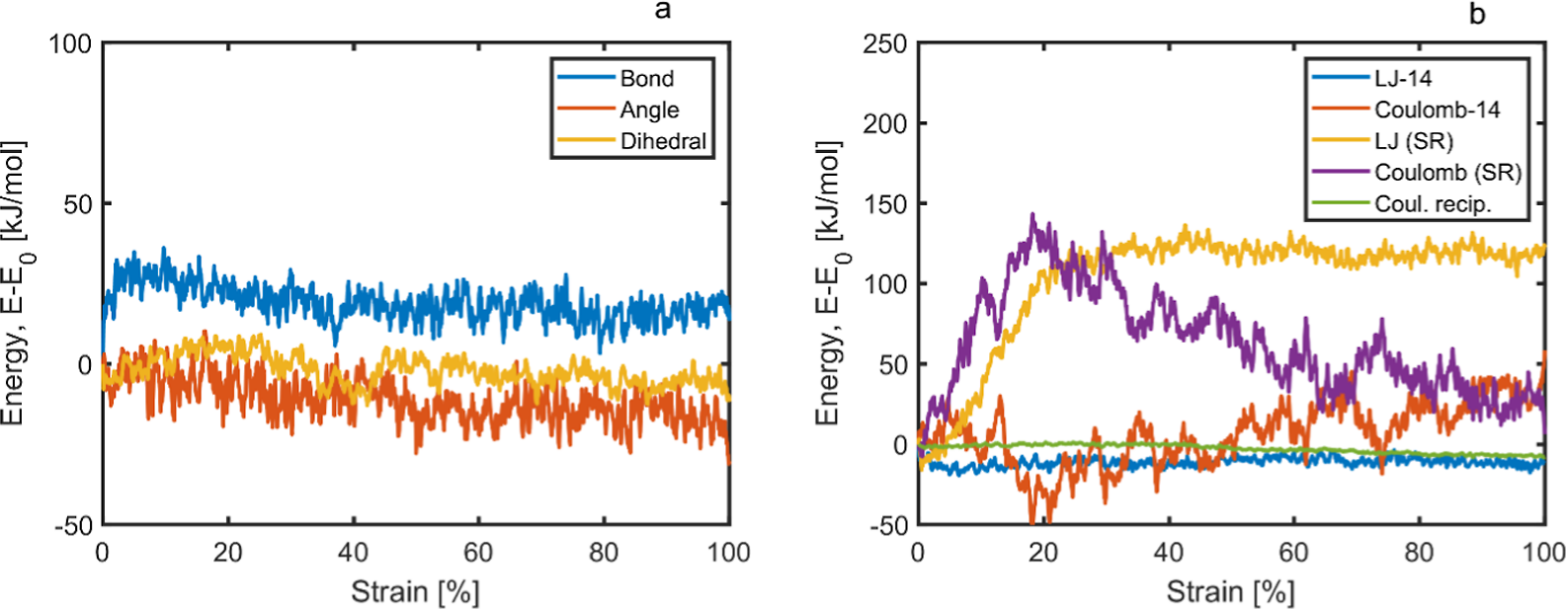
Representative energy (*E* – *E*_0_) plot of cellulose being stretched in one direction, where (a) represents bonded interactions and (b) represents nonbonded interactions.

The main contributing factors to Young’s modulus, Poisson’s ratio, and bulk modulus are revealed when analyzing the energy terms of the stretched samples. An example is depicted in Figure 9, showing energy terms as a function of strain for deformed dry cellulose systems at 300 K. The *y*-axis represents the change in energy (*E* – *E*_0_), where *E* is the energy at a given strain and *E*_0_ is the energy at the start of the simulation (in kJ/mol). Figure 9a shows little change in the energy of the bonds, the bending of the angles, or torsion of the dihedrals as strain increases. In Figure 9b, we observe changes in the Lennard Jones (LJ) and Coulombic contributions, where “14” indicates the atom 1 to atom 4 interactions. Additional examples of energies for deformed polymer systems are presented in Figure SI14. The main contributing factors to (*E* – *E*_0_) are kinetic energies (not shown), which arise from the deformation and facilitate atomic movement, followed by short-range (SR) interactions, Coulombic interactions, and SR LJ interactions. This trend was seen consistently for all the systems, with the only change being in size. Potential energy remained nearly constant for all systems, which is reasonable with view to the fact that no major changes with respect to strain were expected. Kinetic contributions tended to decrease when voids started to form. It was seen that the systems tended to have larger fluctuations the more mobile they became, primarily with increased temperatures but also with higher water contents, as seen in Figure SI14.

## 4 Conclusions

Amorphous cellulose derivatives with hydroxy-, aldehyde-, amine-, and carboxyl-terminated ring openings were simulated using molecular dynamics. The aim was to predict whether the mechanical and thermoplastic properties of cellulose could be enhanced by employing specific ring openings and to gain insight into how thermoplastic cellulose derivatives with improved processability could be achieved. It was observed that ring-opened structures generally yielded more mobile systems compared to structures with unaffected glucose rings. However, ring-opened structures showed significant variation based on the type of modification.

Dialcohol cellulose (23DAC) was one interesting candidate which exhibited a sufficiently low *T*_g_ for processing while maintaining a relatively high Young’s and bulk modulus at 300 K, indicating favorable mechanical properties at room temperature. Another potential candidate is the aminated cellulose system, 23Amin, which was also predicted to possess favorable processing properties (e.g., low *T*_g_, small Young’s modulus, short hydrogen bond half-lives, and high self-diffusivity) at 425 K, along with promising mechanical properties (e.g., high Young’s modulus) at 300 K. Aldehyde systems, such as 23Ald, were projected to offer optimal processing properties at elevated temperatures, albeit with the drawback of high ductility even at room temperature.

Significant differences were seen among the different systems with regard to void formation, which can be related to the tearing or ductility of the systems. With increases in water content and temperature, all systems saw a lower chance of large void formation, which could cause the systems to tear. The systems with aldehyde modification showed the best results in this regard, with the aminated and carboxyl-modified systems being good as well. This may indicate appropriate ductility of these types of systems, but due to the low number of deformation simulations, we cannot state this conclusively.

To conclude, modifications of cellulose can, in an amorphous state, produce derivatives that exhibit significant potential as substitutes for contemporary plastics. Molecular dynamics simulations prove to be an invaluable tool for exploring these new materials. In particular, aldehyde ring openings, if cross-linkages can be avoided, show a low Young’s modulus and a high Poisson’s ratio, making them a good alternative to hard plastics. Dialcohol cellulose may also prove to be a good alternative, as may aminated cellulose ring openings.

## Supporting Information Available

Additional data, system descriptions, simulation details, and additional results, such as energies, densities, and MSD curves (PDF)
