# Supplementary material for: Effects of Ring Opening and Chemical Modification on the Properties of Dry and Moist Cellulose—Predictions with Molecular Dynamics Simulations
Source: Biomacromolecules. 2024 Nov 25;25(12):7581–93. doi: 10.1021/acs.biomac.4c00735 (PMC11632776; doi:10.1021/acs.biomac.4c00735)
Supplement: Supplementary file 1 — bm4c00735_si_001.pdf [file bm4c00735_si_001.pdf]

Supplementary Information

Effects of Ring Opening and Chemical Modification on the Properties of Dry and Moist Cellulose - Predictions with Molecular Dynamics Simulations

Patric Elf[1,3], Per A. Larsson[1,3], Anette Larsson[2,4], Lars Wågberg[1,3], Mikael S. Hedenqvist[1,3*], Fritjof Nilsson[1,5*]

[1]School of Engineering Sciences in Chemistry, Biotechnology and Health,

Fibre and Polymer Technology, KTH Royal Institute of Technology,

SE-100 44 Stockholm, Sweden

[2]Department of Chemistry and Chemical Engineering, Chalmers University of Technology, SE-412 96 Gothenburg, Sweden

[3]FibRe Centre for Lignocellulose-based Thermoplastics, KTH Royal Institute of Technology, Stockholm, Sweden

[4]FibRe Centre for Lignocellulose-based Thermoplastics, Chalmers University of Technology, SE-412 96 Gothenburg, Sweden

[5]FSCN research centre, Mid Sweden University, 85170 Sundsvall, Sweden

Contents

Methodology 2

Tables 3

- Table SI1: Equilibration scheme for the modified 21 step process.. 3
- Table SI2: Normalized volume of simulation box and density of systems.. 4
- Table SI3: Glass transition temperature of systems in Kelvin.. 4
- Table SI4: Drift for PVT curve data, used for T_g calculations.. 5
- Table SI5: Number of water molecules per monomer.. 7
- Table SI6: Hydrogen bonds per monomer at 300K.. 8
- Table SI7: Hydrogen bonds per monomer at 425K.. 9

Figures 10

- Figure SI1: Movement of a single water molecule during the 21-step equilibration of cellulose with 20 wt% water, step 0 to 21.. 10
- Figure SI2: Movement of a single water molecule during the 21-step equilibration of cellulose with 20 wt% water, step 6 to 21.. 11
- Figure SI3: Movement of a single water molecule during the 21-step equilibration of cellulose with 5 wt% water, step 6 to 21.. 11
- Figure SI4: Cellulose during the final step of the 21-step equilibration.. 12
- Figure SI5: Energy during the final step of the 21-step equilibration.. 13
- Figure SI6: Density during the final step of the 21-step equilibration.. 14
- Figure SI7: Energy during the 425K step of T_g simulations.. 15
- Figure SI8: Density during the 425K step of T_g simulations.. 16
- Figure SI9: MSD curves for all systems at 300K.. 17
- Figure SI10: Density for different water contents.. 18
- Figure SI11: Estimated Hydrogen Bond life-times.. 19
- Figure SI12: Total number of hydrogen bonds for at 0, 50 and 100% strain.. 20
- Figure SI13: Free volume over strain for systems not presented in the main article.. 21
- Figure SI14: Energy for a few example systems..22

Methodology

Methodology to evaluate systems is as follows.

Use cellulose (system 1) as a baseline. Look at table SI-1 for guidance.

1. Compare Cellulose and 6Carb for carboxylic acid effect on C6.
2. Compare 23Ald, 23DAC, 23Amin and 23Carb for effect of the different ring openings.
3. Check cumulative effects of carboxylic acid groups using 23Carb and 236Carb
4. Compare 23Ald6Carb and 23DAC6Carb to see if there is a similar effect as in step 1.

Tables

Table SI1: Equilibration scheme for the modified 21 step process. The type represents the type of simulation (NVT or NPT). For NPT, pressure couplings are: B = Berendsen, PR = Parrinello-Rahman, I = Isotropic.

Step	Time [ns]	Type	Pressure [bar]	Temp [K]
0	1	NPT B I	1	500
1	0.5	NVT	-	600
2	0.25	NVT	-	300
3	0.5	NPT B I	99	300
4	0.25	NVT	-	600
5	0.5	NVT	-	300
6	0.5	NPT B I	2961	300
7	0.5	NVT	-	600
8	0.5	NVT	-	300
9	0.5	NPT B I	4935	300
10	0.25	NVT	-	600
11	0.5	NVT	-	300
12	0.5	NPT B I	2467	300
13	0.25	NVT	-	600
14	0.5	NVT	-	300
15	0.25	NPT B I	493	300
16	0.25	NVT	-	600
17	0.5	NVT	-	300
18	0.5	NPT B I	49	300
19	0.25	NVT	-	600
20	0.5	NVT	-	300
21	10	NPT PR I	1	300

Table SI2: Normalized volume of simulation box and density of systems, error estimates/standard deviation are not included as they were less than 1 %. They were all normalized based on their 0 wt% water equivalents.

	Normalized Volume				Normalized Density			
System	0W	5W	10W	20W	0W	5W	10W	20W
Cellulose	1.00	1.04	1.10	1.27	1.00	1.01	1.01	0.98
23Ald	1.00	1.06	1.13	1.31	1.00	1.00	0.98	0.95
23DAC	1.00	1.06	1.12	1.28	1.00	1.00	1.00	0.98
23Amin	1.00	1.06	1.12	1.29	1.00	0.99	0.99	0.97
23Carb	1.00	1.06	1.13	1.32	1.00	0.99	0.98	0.95
6Carb	1.00	1.05	1.11	1.29	1.00	1.01	1.00	0.97
23Ald6Carb	1.00	1.06	1.13	1.31	1.00	1.00	0.99	0.95
23DAC6Carb	1.00	1.05	1.12	1.30	1.00	1.00	0.99	0.96
236Carb	1.00	1.06	1.13	1.33	1.00	1.00	0.98	0.94

Table SI3: Glass transition temperature of systems in Kelvin.

System	0W	5W	10W	20W
Cellulose	444	422	412	404
23Ald	419	384	380	401
23DAC	412	403	396	398
23Amin	407	394	392	400
23Carb	428	396	387	398
6Carb	437	417	396	408
23Ald6Carb	431	389	388	407
23DAC6Carb	428	399	398	400
236Carb	426	408	399	413

Table SI4: Drift for PVT curve data, used for T_g calculations, over the final 0.5 ns of each simulation step. The drift is calculated as the percentage change between the start and end of the sampling interval, divided by the time, giving %/ns.

	Drift in %/ns of the density [kg/m^3] for 0 wt% water systems.							
Temperature	Cellulose	23Ald	23DAC	23Amin	23Carb	6Carb	23Ald6Carb	23DAC6Carb
800	-2.02	0.13	-1.08	0.32	-0.33	3.55	2.05	1.13
775	0.52	0.43	-0.97	-0.20	-0.22	1.85	0.82	1.59
750	-0.62	0.32	-0.76	0.33	-0.42	-0.53	0.10	0.06
725	0.61	-0.98	0.66	-0.31	0.39	-0.71	0.23	1.02
700	-1.13	-0.54	-0.41	-0.68	1.69	0.97	0.26	0.18
675	0.22	0.18	-0.45	0.37	1.28	0.89	0.93	-0.74
650	0.09	0.36	0.69	0.48	0.81	-1.69	0.46	-0.32
625	-0.41	0.87	-0.06	-0.35	0.06	-1.60	0.20	0.58
600	0.31	-0.49	0.97	-0.17	0.62	1.04	-1.42	0.33
575	0.91	0.82	0.25	-1.04	-0.70	0.69	1.36	0.11
550	0.25	-1.87	0.09	0.39	0.22	-0.63	1.13	0.83
525	-0.43	0.81	0.78	0.21	-0.57	-0.99	0.02	0.23
500	0.17	-0.85	-0.87	-1.02	-0.32	0.69	0.06	0.46
475	-0.18	0.34	0.07	-0.31	0.18	-0.17	0.34	-0.55
450	-0.32	-0.53	-0.32	-0.94	0.25	-0.19	0.51	-0.26
425	0.10	0.66	0.03	0.04	-0.09	-0.24	0.27	-0.28
400	-0.27	-0.29	-0.11	-0.15	-0.22	0.13	0.15	0.38
375	0.05	-0.05	-0.08	0.14	-0.01	-0.15	0.28	0.12
350	0.02	-0.22	-0.18	0.08	-0.10	-0.05	-0.07	-0.04
325	-0.02	-0.16	-0.25	0.00	0.15	0.11	-0.10	0.04
300	-0.09	-0.13	-0.25	-0.37	-0.09	0.16	-0.19	-0.17
275	0.07	-0.07	-0.07	-0.09	-0.01	-0.14	-0.02	0.11
250	0.06	0.07	0.03	0.03	-0.06	0.04	0.02	-0.01
225	-0.02	-0.07	-0.14	0.04	-0.06	-0.08	0.06	-0.05
200	0.06	-0.11	0.03	0.02	0.02	0.04	-0.01	-0.01
175	-0.01	0.14	0.01	0.03	0.07	-0.04	0.06	-0.07
150	-0.08	-0.04	-0.05	0.03	-0.04	0.01	0.02	-0.05

	Drift in %/ns of the density [kg/m^3] for 5 wt% water systems.							
Temperature	Cellulose	23Ald	23DAC	23Amin	23Carb	6Carb	23Ald6Carb	23DAC6Carb
600	0.21	0.20	0.28	0.90	-0.57	-1.26	0.77	-0.07
575	-0.93	0.68	-0.23	-0.27	-0.64	-1.34	0.69	0.01
550	0.39	-0.32	0.40	0.87	0.52	-0.11	-0.77	-0.02
525	-0.58	0.58	-0.02	-0.12	0.47	-0.80	-0.69	1.47
500	-0.08	-0.43	0.66	0.05	-0.29	-0.21	-0.46	-0.38
475	0.17	0.51	0.40	0.79	-0.73	0.40	0.98	0.21
450	0.71	-1.05	0.52	-0.24	-0.43	0.09	0.54	0.10
425	-1.01	-0.32	-0.18	0.53	0.84	0.27	0.73	-0.11
400	-0.11	0.19	0.26	0.59	0.59	0.49	-0.08	-0.41
375	0.08	-0.41	-0.43	-0.02	-0.05	-0.05	-0.18	0.13
350	-0.01	0.31	-0.33	0.05	0.04	0.10	-0.29	0.05
325	-0.09	-0.16	-0.14	-0.15	-0.10	0.18	0.06	0.20

300	-0.03	0.07	-0.26	-0.05	0.10	-0.05	0.13	0.30
275	-0.01	0.15	-0.21	-0.07	-0.03	-0.07	0.09	0.17
250	-0.17	0.24	-0.06	0.06	0.10	-0.11	0.19	0.01
225	0.12	0.04	-0.02	-0.11	-0.01	0.13	0.12	0.06
200	-0.04	-0.17	0.05	-0.07	0.04	0.05	-0.21	-0.09
175	-0.03	-0.01	0.09	-0.03	0.06	-0.05	0.01	0.10
150	0.02	-0.01	0.03	0.01	-0.05	-0.03	0.03	-0.06

Drift in %/ns of the density [kg/m^3] for 10 wt% water systems.

Temperature	Cellulose	23Ald	23DAC	23Amin	23Carb	6Carb	23Ald6Carb	23DAC6Carb
600	0.02	1.47	0.11	0.06	0.77	-0.59	-1.36	-0.46
575	-0.73	-0.44	-0.60	-1.40	0.55	-1.01	0.66	-0.82
550	-1.28	0.02	-0.09	-0.32	-0.62	0.37	-1.38	-0.71
525	0.19	0.71	-0.33	0.13	-0.26	0.17	0.36	0.09
500	0.19	0.61	0.35	-0.21	0.12	-0.02	-0.66	0.06
475	0.49	0.40	-0.61	0.18	-0.07	-0.30	-0.61	0.03
450	-0.06	0.36	-0.28	0.08	0.09	0.08	0.39	0.06
425	-0.07	0.02	0.48	0.38	-0.45	-0.41	0.51	-0.90
400	0.30	0.56	-0.50	0.23	0.06	-0.06	-1.03	0.09
375	-0.19	0.24	-0.23	0.19	0.15	-0.09	0.05	0.35
350	-0.35	-0.10	0.36	0.08	0.09	-0.12	-0.25	-0.55
325	-0.18	0.18	0.57	0.35	0.17	-0.11	0.39	0.09
300	0.18	-0.03	0.18	-0.08	-0.22	-0.25	-0.42	-0.16
275	0.02	0.00	0.29	-0.14	-0.16	-0.01	-0.24	0.27
250	0.05	-0.06	-0.10	-0.03	0.11	0.21	0.09	-0.13
225	0.04	-0.22	0.02	0.00	-0.01	0.04	0.02	-0.11
200	0.15	0.07	-0.05	-0.09	-0.10	0.01	-0.09	0.00
175	0.02	-0.08	0.03	0.09	-0.04	0.06	0.01	-0.06
150	0.05	-0.09	-0.04	0.04	0.03	-0.03	0.03	0.03

Drift in %/ns of the density [kg/m^3] for 20 wt% water systems.

Temperature	Cellulose	23Ald	23DAC	23Amin	23Carb	6Carb	23Ald6Carb	23DAC6Carb
600	-1.14	-0.06	0.72	-0.57	1.43	0.04	-0.07	-1.53
575	-1.05	-0.86	0.30	-0.21	0.59	0.47	-0.92	0.42
550	-0.37	0.01	0.34	-0.62	0.68	-0.04	-0.13	-0.39
525	0.12	-0.23	0.28	-0.39	0.30	0.24	-0.19	0.03
500	0.08	-0.41	-0.47	-0.64	0.29	-0.01	0.02	-0.24
475	-0.19	-0.12	-0.07	-0.41	-0.39	-0.58	0.66	-0.07
450	0.20	-0.13	-0.11	0.01	-0.63	-0.22	0.03	0.61
425	1.24	-0.27	-0.14	1.03	-0.23	-0.28	0.21	-0.02
400	0.64	0.59	0.85	0.33	-0.07	0.62	-0.04	0.30
375	-0.55	0.39	0.20	0.21	-0.06	-0.18	0.60	-0.18
350	-0.18	-0.19	0.00	-0.03	0.23	0.00	0.70	-0.34
325	-0.01	-0.30	0.11	0.12	-0.03	-0.41	-0.31	0.00
300	-0.07	0.35	0.00	0.40	0.00	-0.48	-0.02	0.10
275	0.13	-0.41	0.52	0.33	0.17	0.43	-0.21	0.09
250	-0.20	-0.11	-0.04	-0.14	0.16	-0.06	0.06	-0.26
225	-0.10	0.10	-0.12	0.25	0.11	-0.28	-0.14	-0.10

200	0.01	0.19	-0.07	-0.01	0.12	-0.07	-0.12	-0.08
175	0.10	-0.03	-0.12	-0.03	-0.12	-0.02	-0.16	-0.06
150	-0.06	-0.04	0.01	-0.06	-0.03	0.00	-0.08	-0.01

Table SI5: Number of water molecules per monomer.

System	20W	10W	5W
Cellulose	2.24	1.00	0.47
23Ald	2.21	0.98	0.47
23DAC	2.27	1.01	0.48
23Amin	2.68	1.19	0.56
23Carb	2.65	1.18	0.56
6Carb	2.43	1.08	0.51
23Ald6Carb	2.40	1.07	0.51
23DAC6Carb	2.46	1.09	0.52
236Carb	2.85	1.26	0.60

Table SI6: Hydrogen bonds per monomer at 300K.

Cellulose	0W	5W	10W	20W
Poly-Poly	2.22±0.02	1.98±0.02	1.77±0.02	1.48±0.02
Poly-Water		1.04±0.02	1.85±0.02	2.81±0.03
Water-Water		0.17±0.01	0.58±0.01	2.11±0.02
**23Ald**	**0W**	**5W**	**10W**	**20W**
Poly-Poly	0.81±0.01	0.64±0.01	0.52±0.01	0.37±0.01
Poly-Water		0.79±0.02	1.33±0.02	2.00±0.03
Water-Water		0.19±0.01	0.65±0.01	2.21±0.02
**23DAC**	**0W**	**5W**	**10W**	**20W**
Poly-Poly	2.51±0.02	2.16±0.02	1.90±0.02	1.49±0.02
Poly-Water		1.14±0.02	1.95±0.02	3.18±0.03
Water-Water		0.14±0.01	0.56±0.01	1.99±0.02
**23Amin**	**0W**	**5W**	**10W**	**20W**
Poly-Poly	3.90±0.03	3.41±0.03	3.00±0.03	2.43±0.03
Poly-Water		1.48±0.02	2.73±0.03	4.39±0.05
Water-Water		0.20±0.01	0.65±0.01	2.33±0.02
**23Carb**	**0W**	**5W**	**10W**	**20W**
Poly-Poly	1.99±0.02	1.70±0.02	1.47±0.02	1.13±0.02
Poly-Water		1.11±0.02	1.90±0.02	2.91±0.03
Water-Water		0.22±0.01	0.78±0.01	2.64±0.02
**6Carb**	**0W**	**5W**	**10W**	**20W**
Poly-Poly	1.97±0.02	1.76±0.02	1.53±0.02	1.21±0.02
Poly-Water		1.04±0.02	1.81±0.02	2.95±0.03
Water-Water		0.21±0.01	0.71±0.01	2.31±0.02
**23Ald6Carb**	**0W**	**5W**	**10W**	**20W**
Poly-Poly	0.63±0.01	0.56±0.01	0.48±0.01	0.39±0.01
Poly-Water		0.68±0.01	1.19±0.02	1.83±0.03
Water-Water		0.23±0.01	0.74±0.01	2.44±0.02
**23DAC6Carb**	**0W**	**5W**	**10W**	**20W**
Poly-Poly	2.15±0.02	1.83±0.02	1.59±0.02	1.18±0.02
Poly-Water		1.13±0.02	1.90±0.02	3.10±0.03
Water-Water		0.20±0.01	0.70±0.01	2.32±0.02
**236Carb**	**0W**	**5W**	**10W**	**20W**
Poly-Poly	1.84±0.02	1.62±0.02	1.45±0.02	1.13±0.02
Poly-Water		1.04±0.02	1.77±0.02	2.81±0.03
Water-Water		0.26±0.01	0.88±0.01	2.86±0.03

Table SI7: Hydrogen bonds per monomer at 425K.

Cellulose	0W	5W	10W	20W
Poly-Poly	2.03±0.02	1.81±0.02	1.63±0.02	1.38±0.02
Poly-Water		0.86±0.02	1.45±0.03	2.17±0.04
Water-Water		0.13±0.01	0.49±0.02	1.77±0.03
23Ald	0W	5W	10W	20W
Poly-Poly	0.71±0.01	0.56±0.01	0.46±0.01	0.33±0.01
Poly-Water		0.64±0.02	1.07±0.03	1.66±0.03
Water-Water		0.14±0.01	0.51±0.02	1.69±0.03
23DAC	0W	5W	10W	20W
Poly-Poly	2.20±0.02	1.90±0.02	1.66±0.02	1.32±0.02
Poly-Water		0.91±0.02	1.56±0.03	2.44±0.04
Water-Water		0.13±0.01	0.47±0.02	1.67±0.03
23Amin	0W	5W	10W	20W
Poly-Poly	3.21±0.04	2.79±0.04	2.46±0.04	1.98±0.04
Poly-Water		1.19±0.03	2.06±0.04	3.29±0.05
Water-Water		0.15±0.01	0.54±0.02	1.93±0.03
23Carb	0W	5W	10W	20W
Poly-Poly	1.76±0.02	1.45±0.02	1.23±0.02	0.90±0.02
Poly-Water		0.94±0.02	1.57±0.03	2.43±0.04
Water-Water		0.17±0.01	0.62±0.02	2.09±0.03
6Carb	0W	5W	10W	20W
Poly-Poly	1.85±0.02	1.57±0.02	1.36±0.02	1.10±0.02
Poly-Water		0.90±0.02	1.55±0.03	2.31±0.04
Water-Water		0.15±0.01	0.54±0.02	1.94±0.03
23Ald6Carb	0W	5W	10W	20W
Poly-Poly	0.56±0.01	0.47±0.01	0.40±0.01	0.30±0.01
Poly-Water		0.58±0.02	1.00±0.02	1.54±0.03
Water-Water		0.17±0.01	0.57±0.02	1.92±0.03
23DAC6Carb	0W	5W	10W	20W
Poly-Poly	1.87±0.02	1.56±0.02	1.32±0.02	0.99±0.02
Poly-Water		0.92±0.02	1.59±0.03	2.49±0.04
Water-Water		0.15±0.01	0.54±0.02	1.88±0.03
236Carb	0W	5W	10W	20W
Poly-Poly	1.63±0.02	1.43±0.02	1.21±0.02	0.91±0.02
Poly-Water		0.87±0.02	1.49±0.03	2.26±0.04
Water-Water		0.20±0.01	0.70±0.02	2.35±0.03

Figures

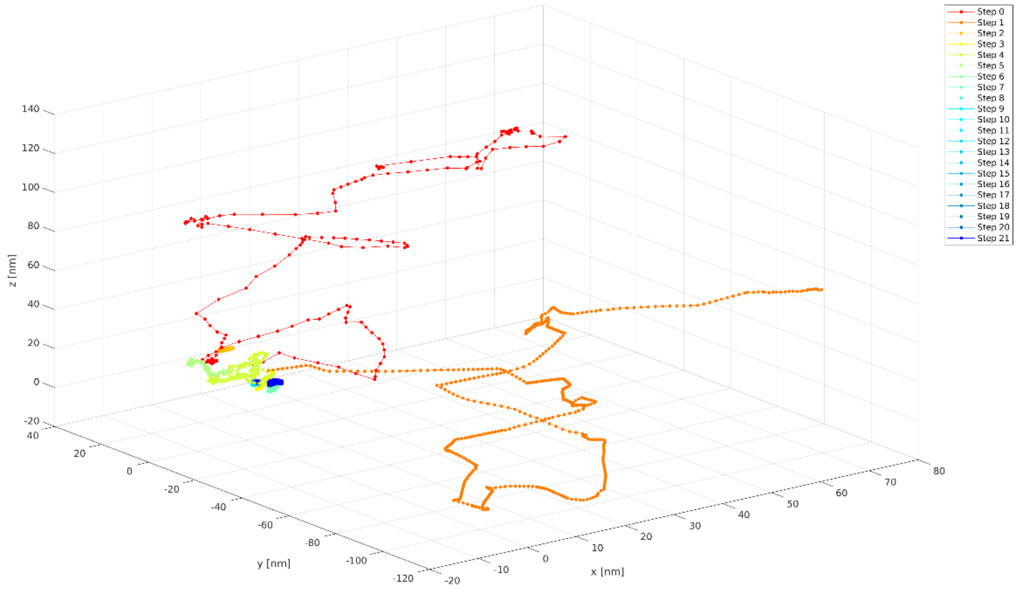

Figure SI1: Movement of a single water molecule during the 21-step equilibration of cellulose with 20 wt% water, step 0 to 21.

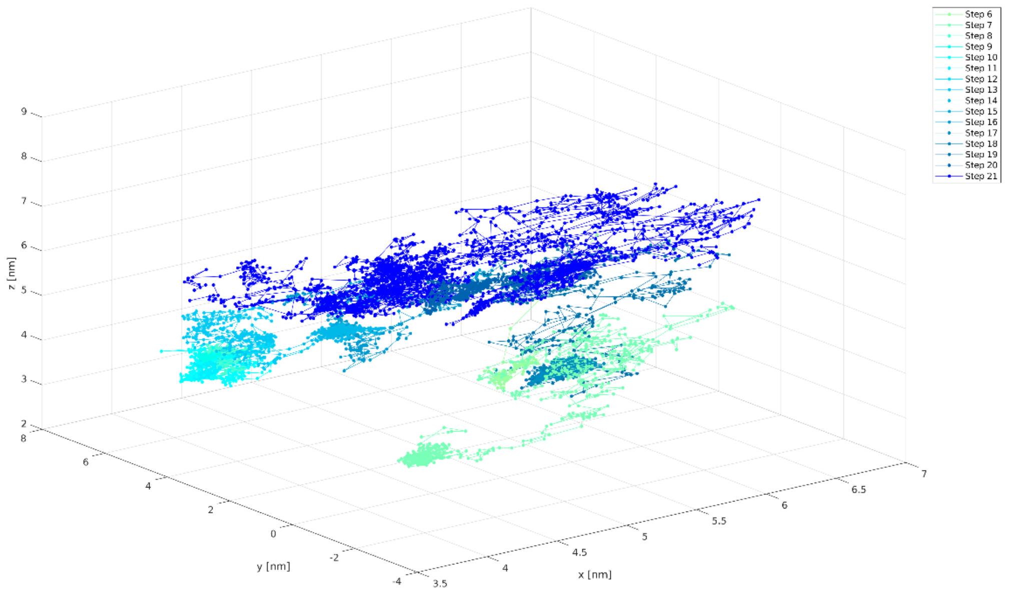

Figure SI2: Movement of a single water molecule during the 21-step equilibration of cellulose with 20 wt% water, step 6 to 21.

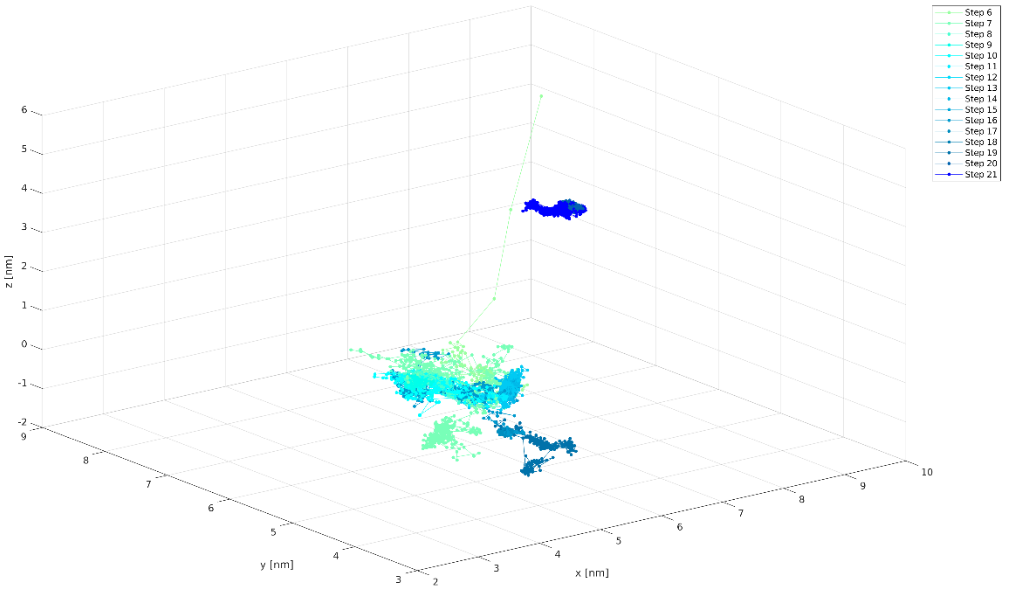

Figure SI3: Movement of a single water molecule during the 21-step equilibration of cellulose with 5 wt% water, step 6 to 21.

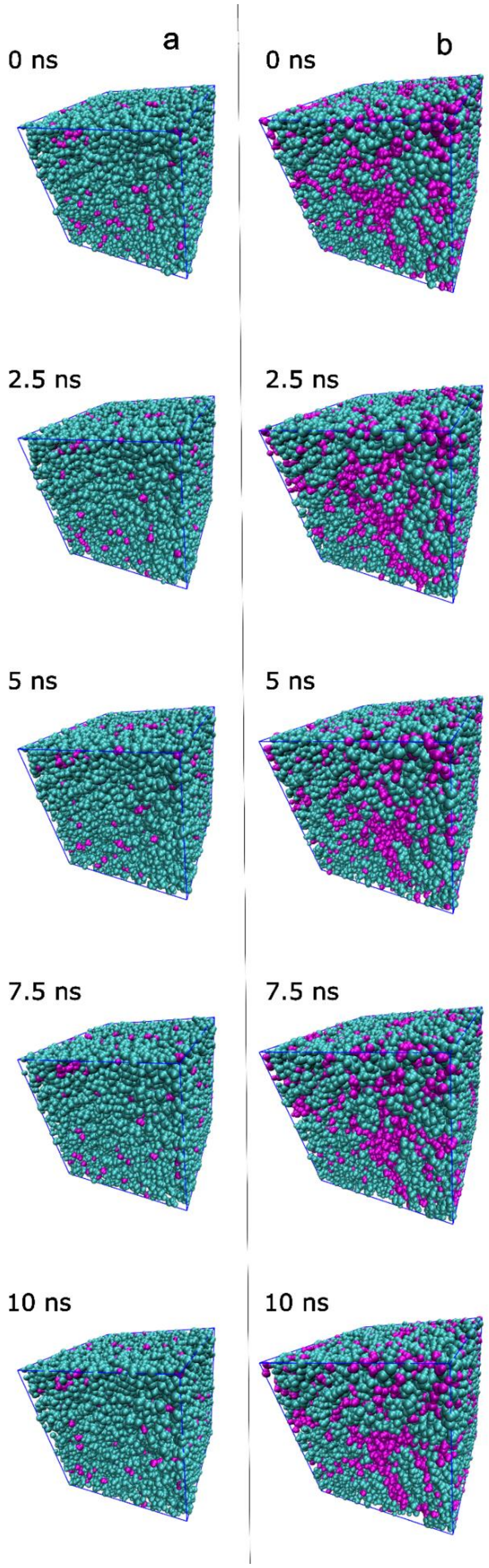

Figure SI4: Cellulose with a) 5 wt% water and b) 20 wt% water (right) during the final step of the 21-step equilibration, with snapshots at 0, 2.5, 5, 7.5 and 10 ns.

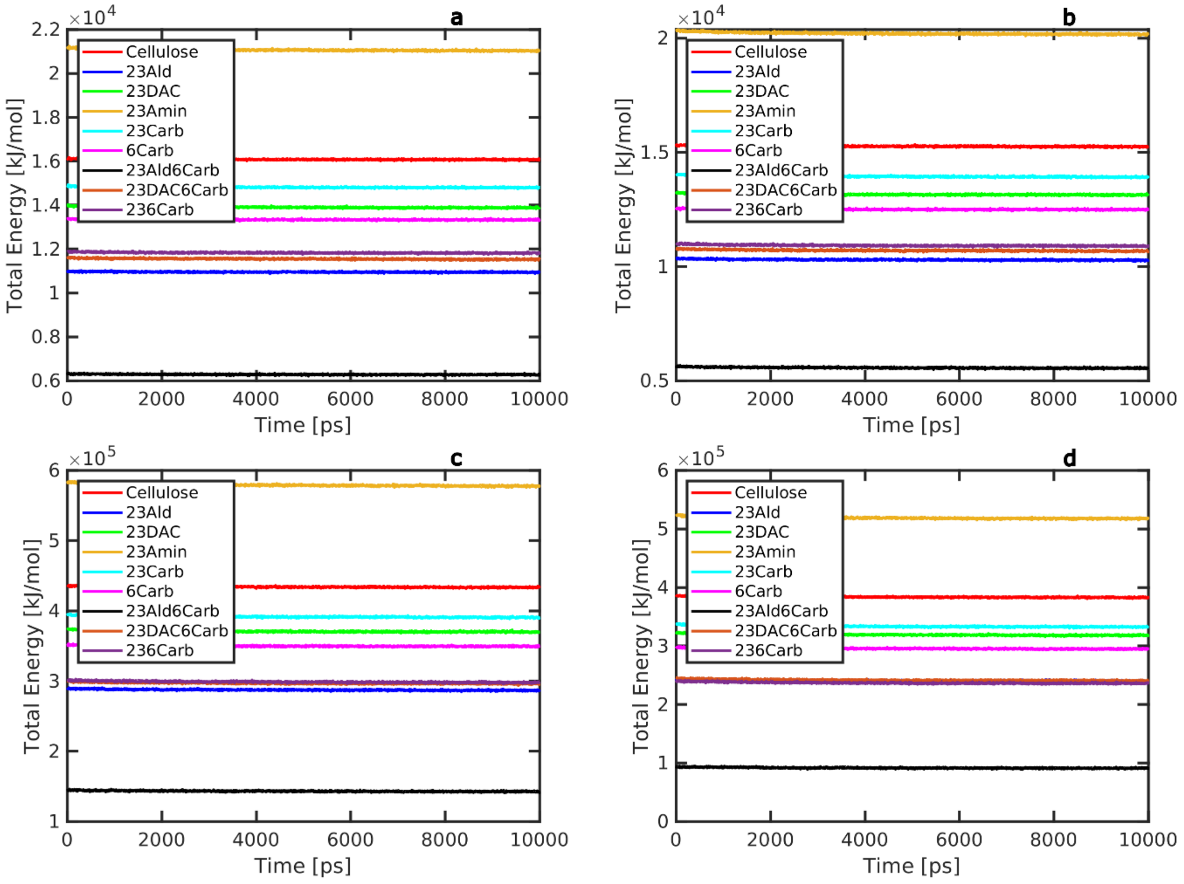

Figure SI5: Energy during the final step of the 21-step equilibration for: a) 300K and 0 wt% water, b) 300K and 5 wt% water, c) 300K and 10 wt% water and d) 300K and 20 wt% water.

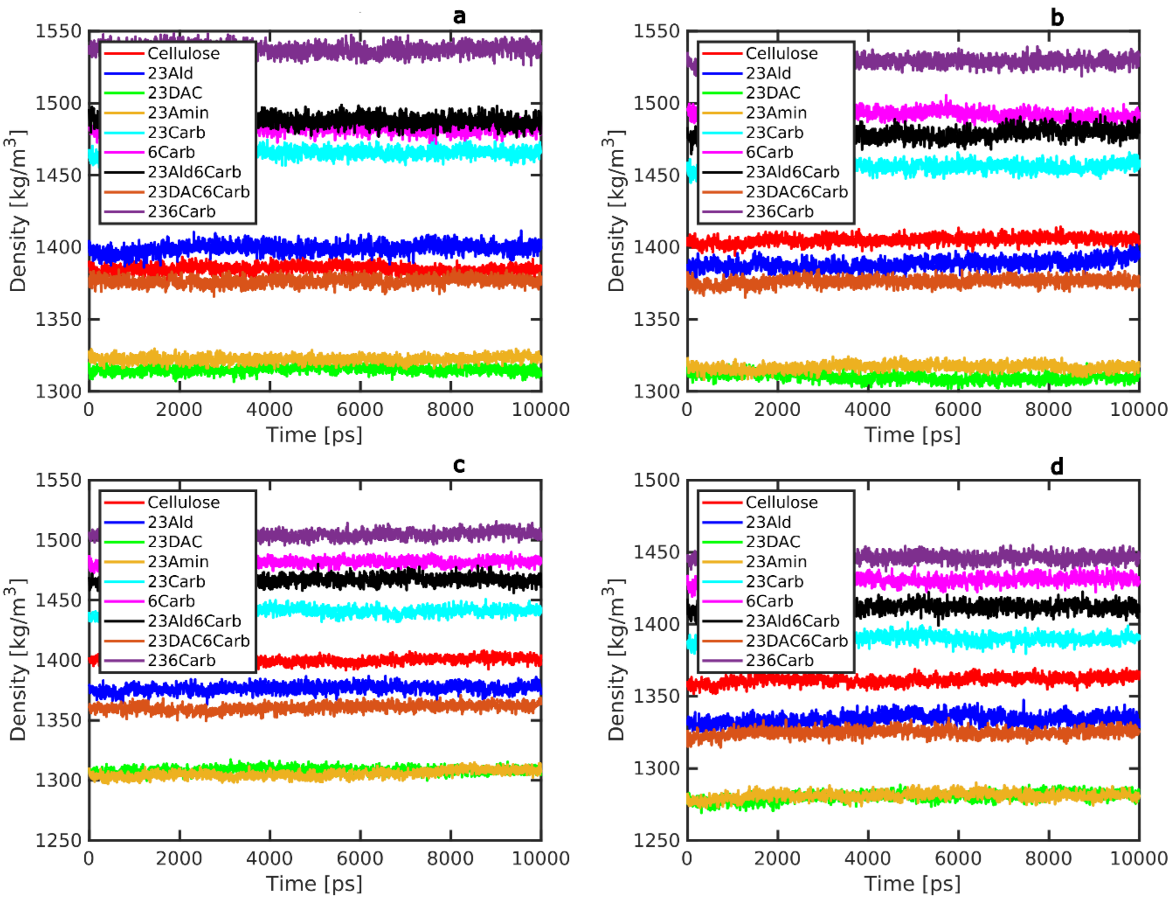

Figure SI6: Density during the final step of the 21-step equilibration for: a) 300K and 0 wt% water, b) 300K and 5 wt% water, c) 300K and 10 wt% water and d) 300K and 20 wt% water.

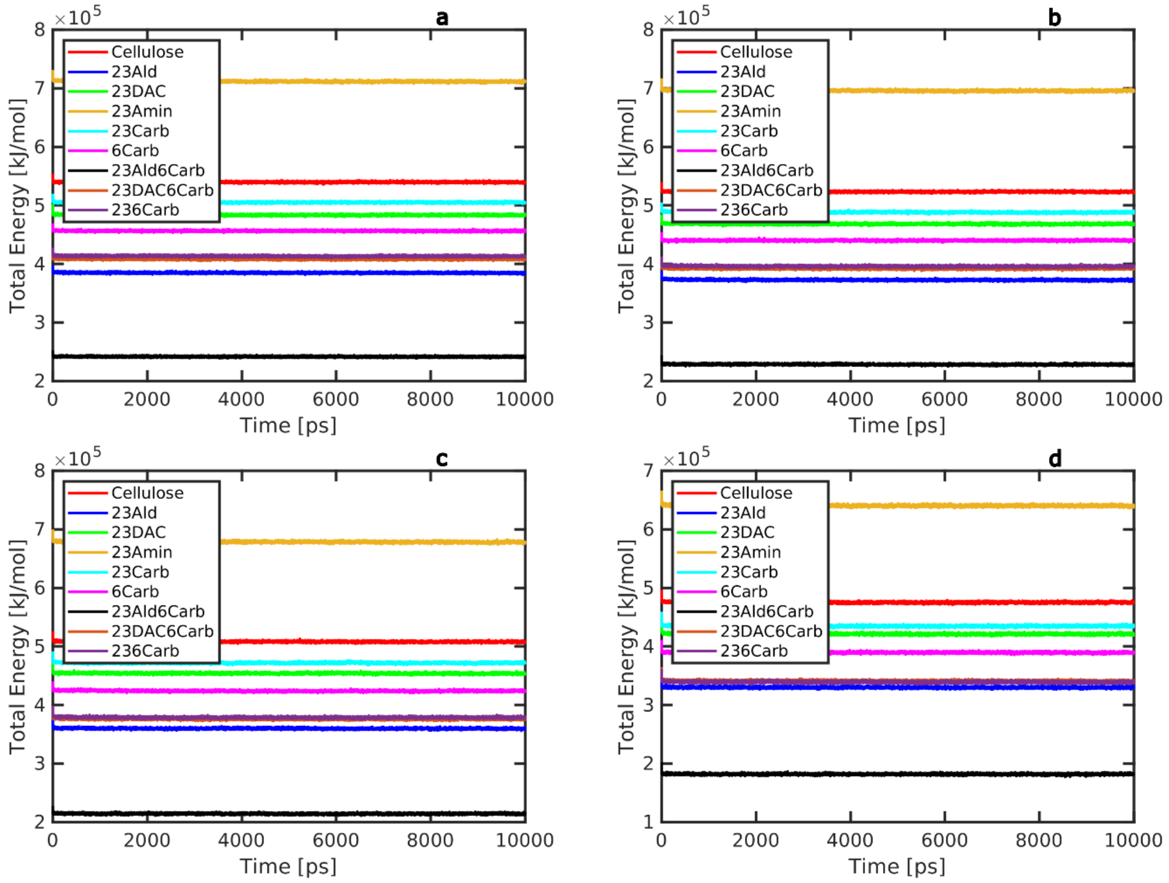

Figure SI7: Energy during the 425K step of T_g simulations for: a) 425K and 0 wt% water, b) 425K and 5 wt% water, c) 425K and 10 wt% water and d) 425K and 20 wt% water.

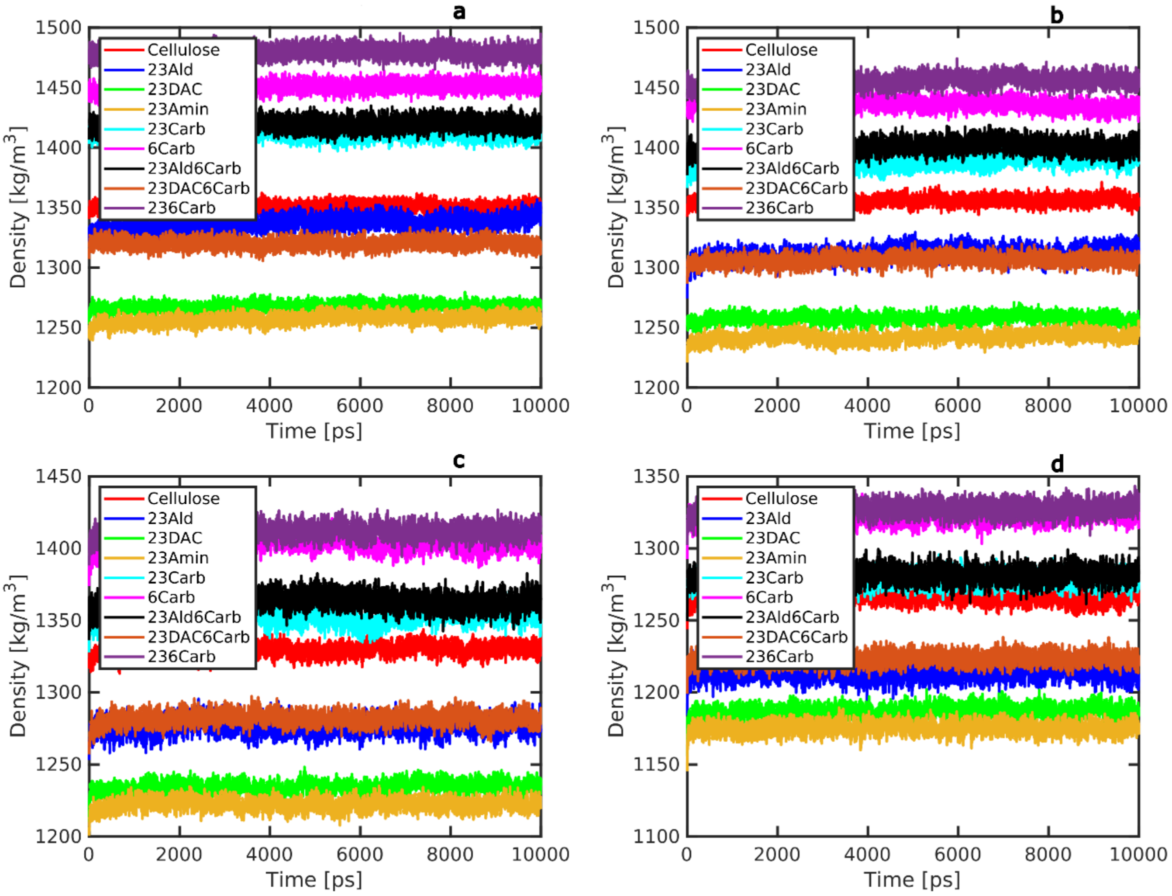

Figure SI8: Density during the 425K step of T_g simulations for: a) 425K and 0 wt% water, b) 425K and 5 wt% water, c) 425K and 10 wt% water and d) 425K and 20 wt% water.

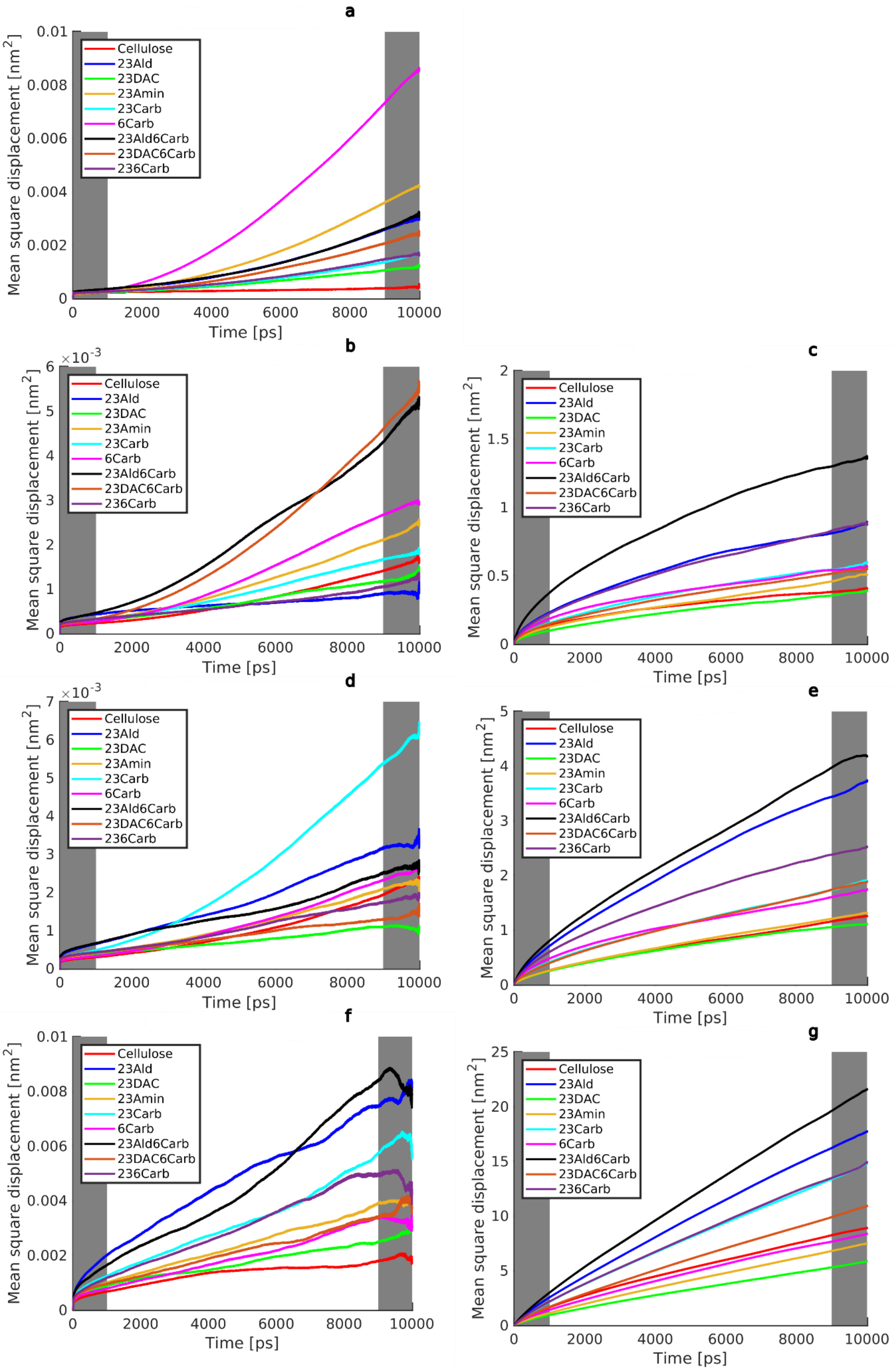

Figure SI9: MSD curves for all systems at 300K. Diffusivity was calculated for the curves within the white background, meaning between 1000 and 9000 ps. a) Polymers at 300K and 0 wt% water, b) Polymer at 300K and 5 wt% water, c) water at 300K and 5 wt% water, d) Polymer at 300K and 10 wt% water, e) Water at 300K and 10 wt% water, f) Polymer at 300K and 20 wt% water, g) Water at 300K and 20 wt% water.

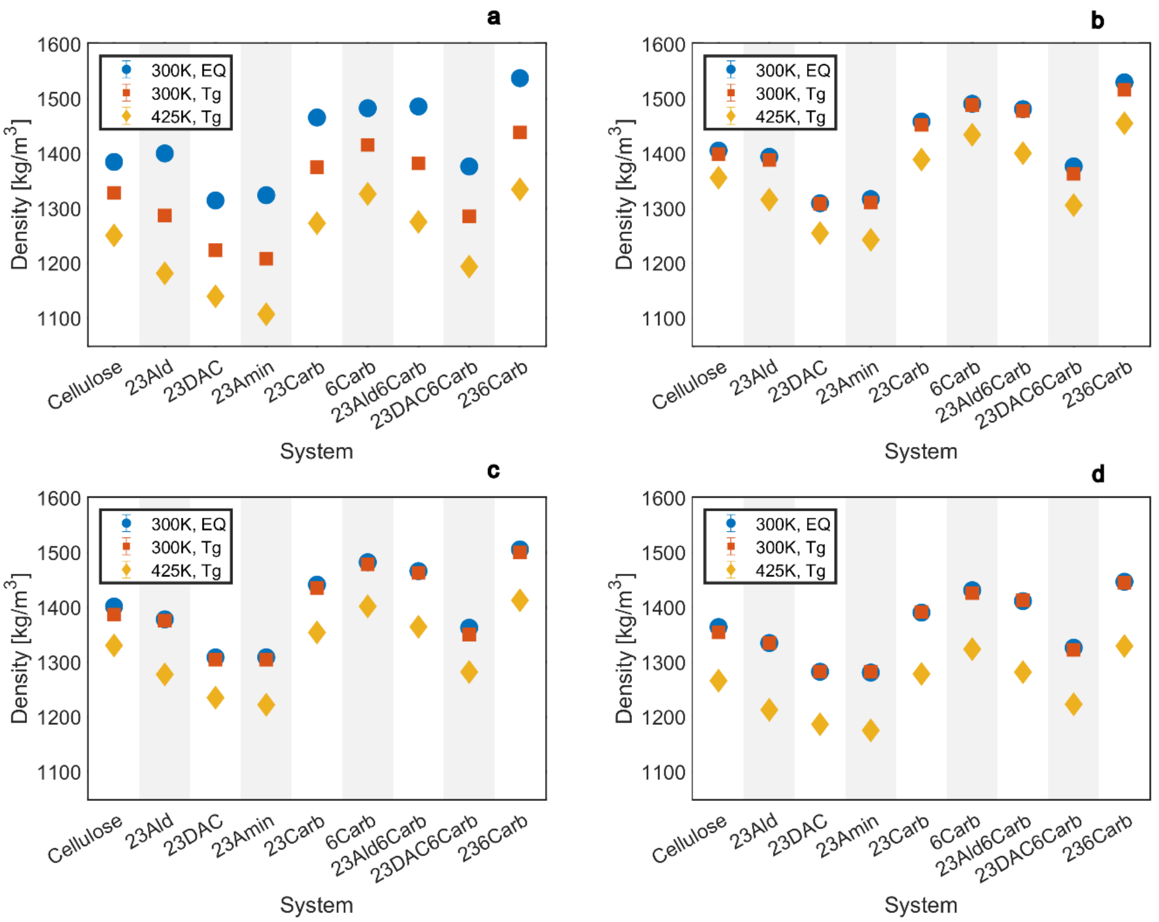

Figure SI10: Density for different water contents. EQ in this case, indicates that the results were from the final 0.5 ns of the last step of the 21-step equilibration, while T_g indicates the measurements were taken from the temperature step of the T_g NVT simulations. a) 0 wt% water, b) 5 wt% water, c) 10 wt% water, d) 20 wt% water.

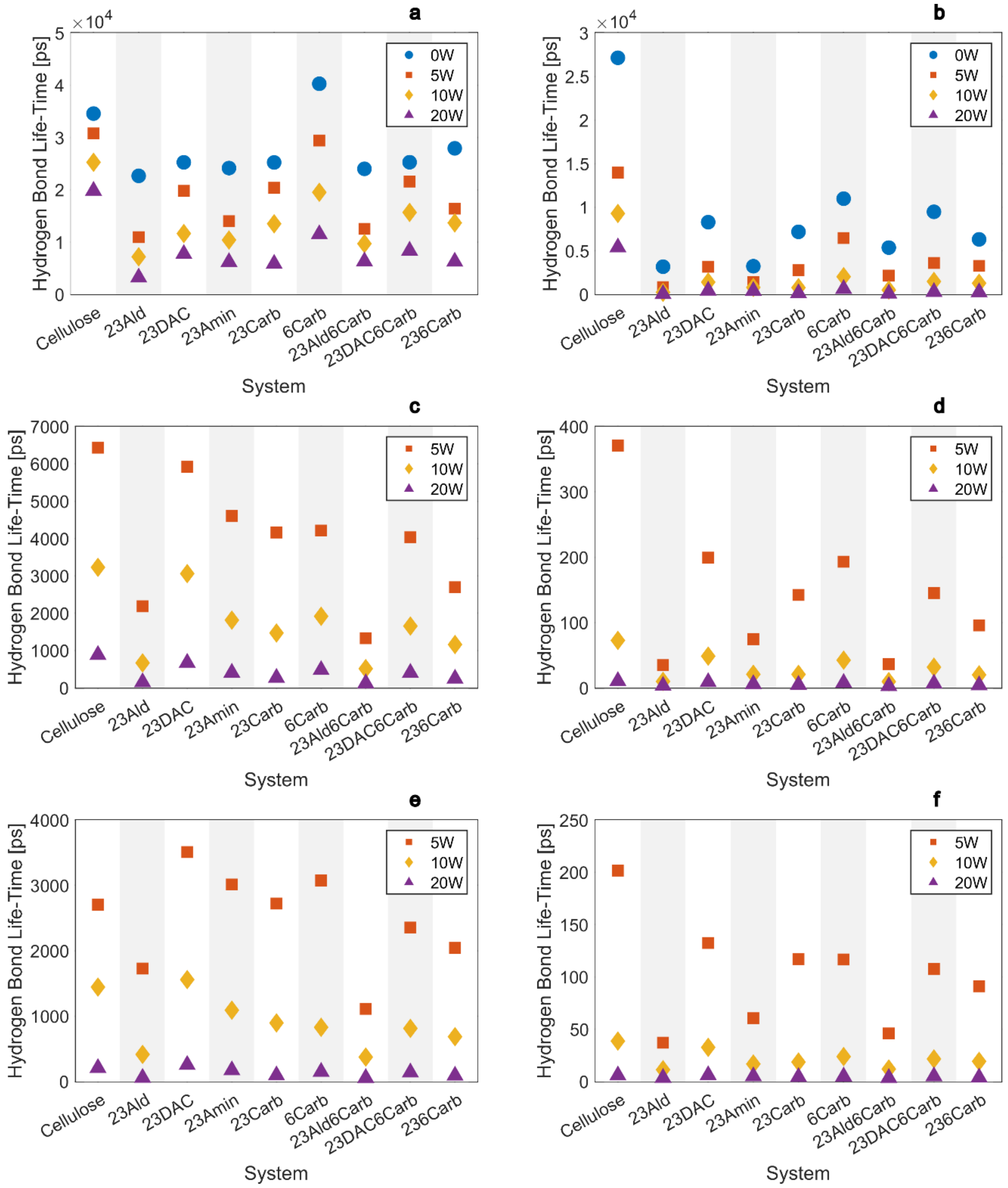

Figure SI11: Estimated Hydrogen Bond life-times for a) Polymer-Polymer at 300K, b) Polymer-Polymer at 425K, c) Polymer-Water at 300K, d) Polymer-Water at 425K, e) Water-Water at 300K, f) Water-Water at 425K.

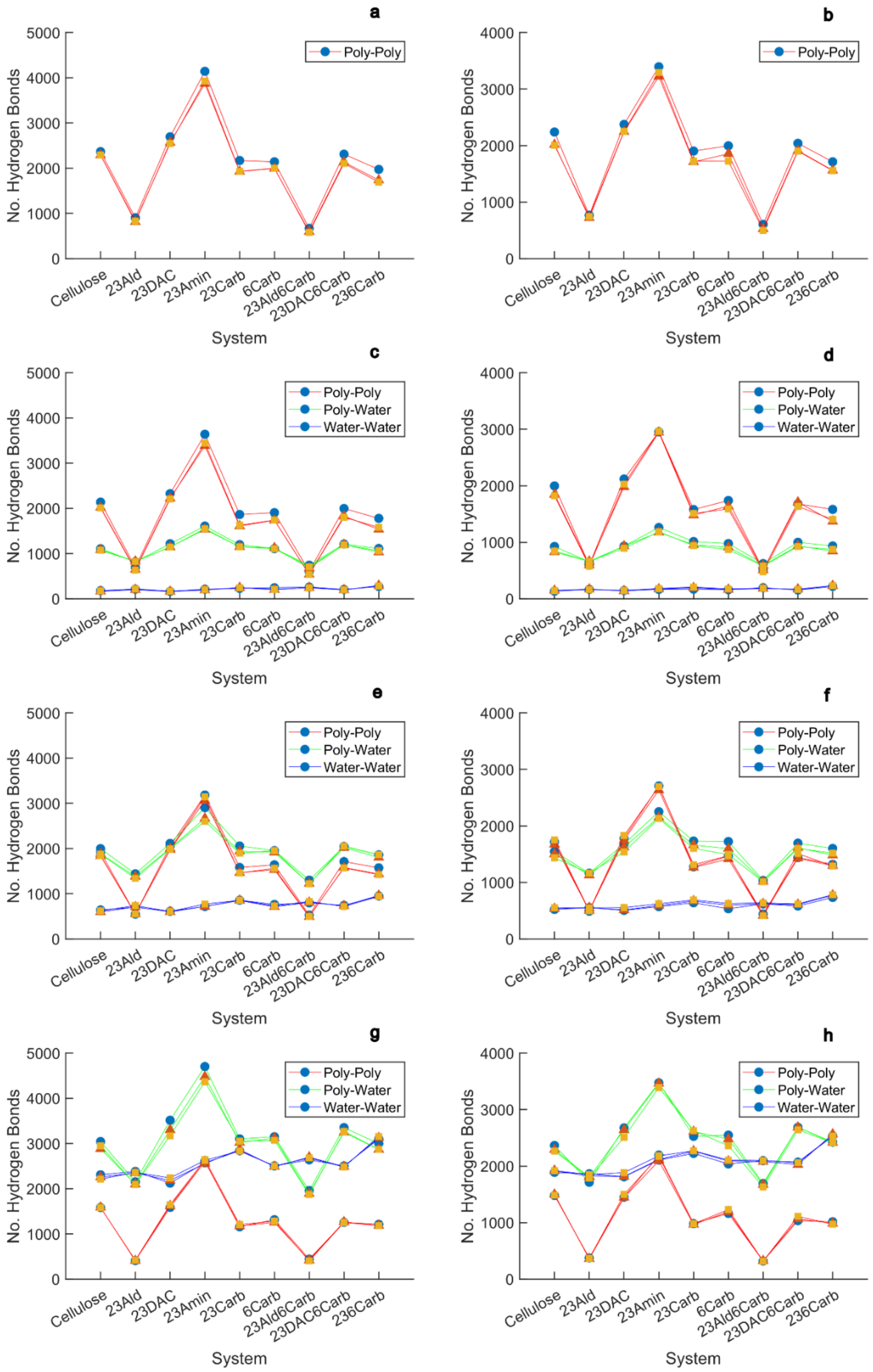

Figure SI12: Total number of hydrogen bonds for a) 300K and 0 wt% water, b) 425K and 0 wt% water, c) 300K and 5 wt% water, d) 425K and 5 wt% water, e) 300K and 10 wt% water, f) 425K and 10 wt% water, g) 300K and 20 wt% water and h) 425K and 20 wt% water. Lines to help guide between the different types of hydrogen bonds. Blue circle is at 0% strain, orange triangle is at 50% strain and yellow square is at 100% strain.

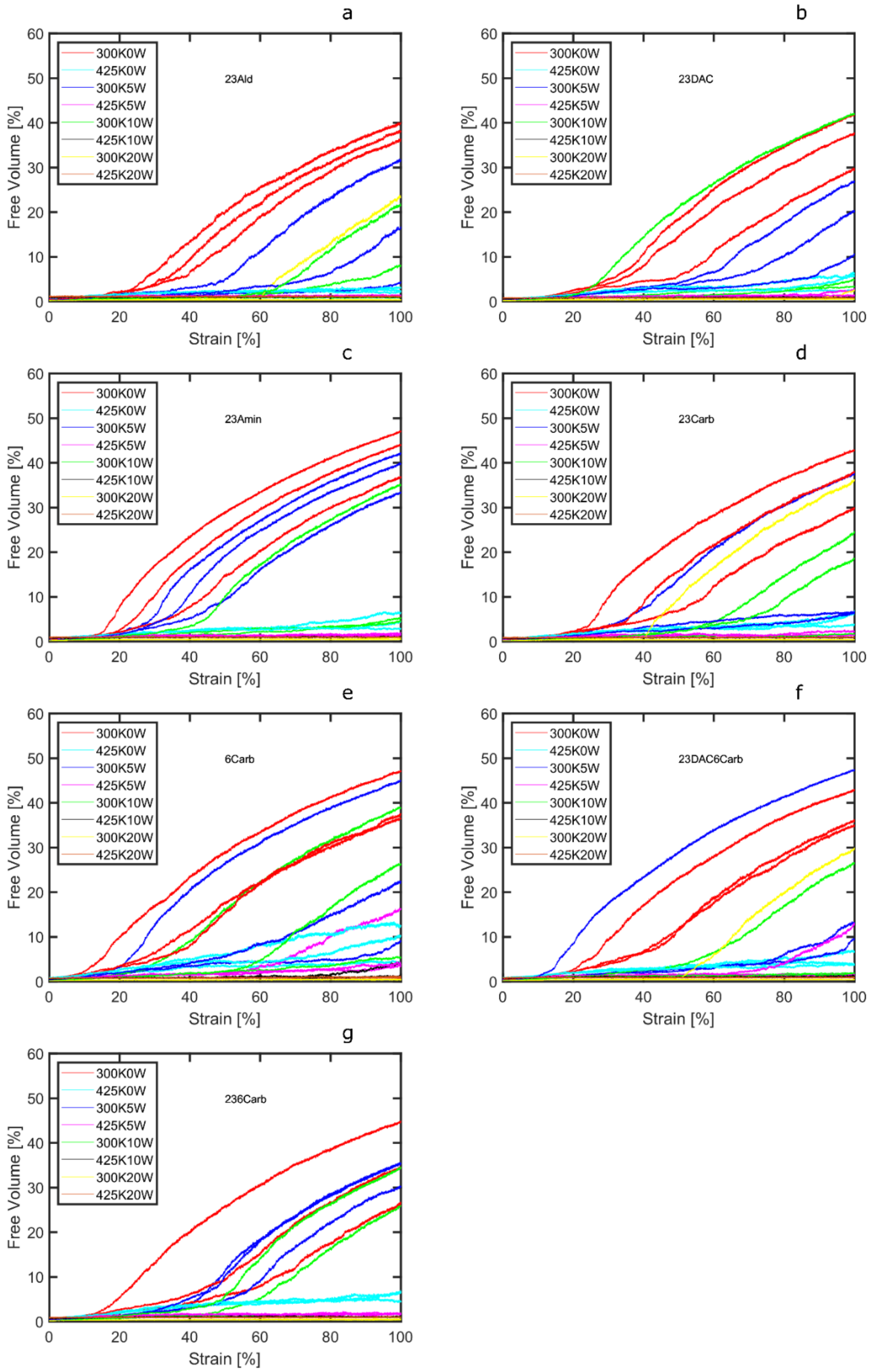

Figure SI13: Free volume over strain for systems not already presented in the main article, using 0.1 nm probe size. The systems were strained in 3 directions, meaning each temperature and water content are presented trice, for: a) 23Ald, b) 23DAC, c) 23Amin, d) 23Carb, e) 6Carb, f) 23DAC6Carb, g) 236Carb. XKYW indicates X Temperature and Y wt% water. As an example, 300K5W means 300 K and 5 wt% water.

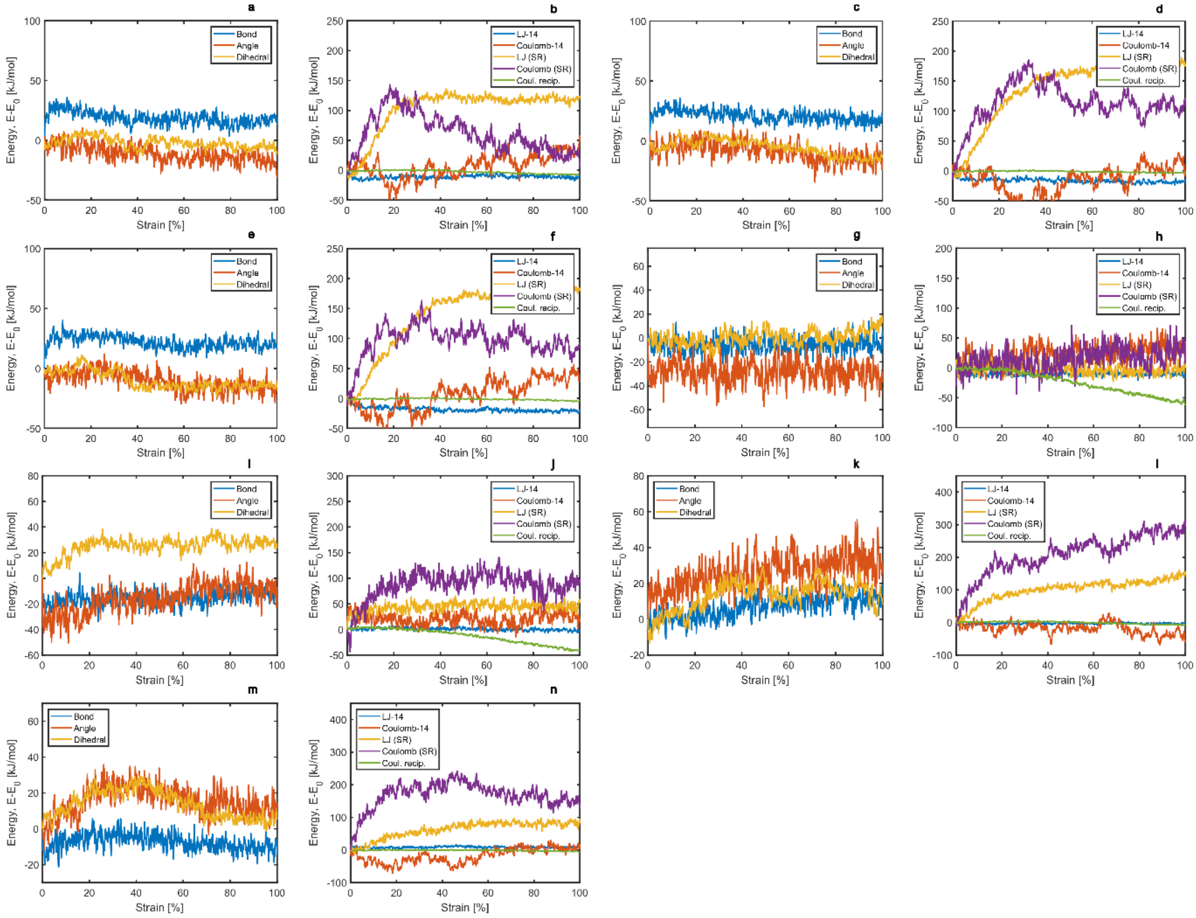

Figure SI14: Energy for a few example systems, for Cellulose at 0 wt% water in a) x-direction, bonded, b) x-direction non-bonded, c) y-direction bonded d) y-direction non-bonded, e) z-direction bonded, f) z-direction non-bonded, for the 236Carb system at 425K and 20 wt% water in g) z-direction bonded, h) z-direction non-bonded, for 236Carb system at 300K and 20 wt% water in i) z-direction bonded, j) z-direction non-bonded, for 23Amin system at 300K and 10 wt% water in k) x-direction bonded, l) x-direction non-bonded, for Cellulose at 300K and 20 wt% water in m) z-direction bonded, n) z-direction non-bonded.
